# Mathematical analysis of phototransduction reaction parameters in rods and cones

**DOI:** 10.1038/s41598-022-23069-0

**Published:** 2022-11-14

**Authors:** Yukari Takeda, Kazuma Sato, Yukari Hosoki, Shuji Tachibanaki, Chieko Koike, Akira Amano

**Affiliations:** 1 Department of Integrative & Systems Physiology, Faculty of Medical Sciences, University of Fukui, Fukui, Fukui Japan; 2 Department of Life Sciences, Ritsumeikan University, 1-1-1 Nojihigashi, Kusatsu, Shiga 525-8577 Japan; 3 Graduate School of Frontier Biosciences, Osaka University, Suita, Osaka Japan; 4 Department of Pharmaceutical Sciences, Ritsumeikan University, Kusatsu, Shiga Japan

**Keywords:** Computational models, Neurophysiology, Biochemistry, Cell biology, Neuroscience

## Abstract

Retinal photoreceptor cells, rods and cones, convert photons of light into chemical and electrical signals as the first step of the visual transduction cascade. Although the chemical processes in the phototransduction system are very similar to each other in these photoreceptors, the light sensitivity and time resolution of the photoresponse in rods are functionally different than those in the photoresponses of cones. To systematically investigate how photoresponses are divergently regulated in rods and cones, we have developed a detailed mathematical model on the basis of the Hamer model. The current model successfully reconstructed light intensity-, ATP- and GTP-dependent changes in concentrations of phosphorylated visual pigments (VPs), activated transducins (Tr*s) and phosphodiesterases (PDEs) in rods and cones. In comparison to rods, the lower light sensitivity of cones was attributed not only to the lower affinity of activated VPs for Trs but also to the faster desensitization of the VPs. The assumption of an intermediate inactive state, MII^i^, in the thermal decay of activated VPs was essential for inducing faster inactivation of VPs in rods, and possibly also in cones.

## Introduction

Retinal photoreceptor cells, the key players in the visual system, convert photons of light into chemical and electrical signals as the first step of the visual transduction cascade. When incident light stimulates a visual pigment (VP; a prototypical G protein-coupled receptor) on membranous disks at the outer segments of photoreceptors, the catalytic activity of a heterotrimeric G-protein, transducin (Tr), of exchanging GTPs for previously bound GDP increases. The GTP-bound α subunit of Tr dissociates from the βγ subunits and subsequently activates phosphodiesterase (PDE)^1^, which in turn hydrolyzes cyclic guanosine 3′–5′ monophosphate (cGMP). The signals of visual transduction are highly amplifiable since a single stimulated VP (VP*) activates ~ 30 and ~ 140 molecules of Tr per second in cones and rods, respectively^2^. Activated VPs are simultaneously inactivated through two distinct mechanisms: phosphorylation by visual pigment-specific kinases (RKs) and thermal decay processes, thereby contributing to the termination of Tr activity.

Under scotopic conditions (dark), basal cGMP regulates the activities of cyclic nucleotide-gated (CNG) nonselective cation channels, allowing a steady inward current^3^. Photoreceptor cells are thus slightly depolarized (− 40 mV^4^) and spontaneously release a neurotransmitter, glutamate^5^. Upon stimulation of visual pigments the concentration of cGMP ([cGMP]) decreases, which deactivates the CNG channels and subsequently hyperpolarizes the photoreceptor to ~ − 60 mV, preventing the release of neurotransmitters^4^. Postsynaptic responses to glutamate may be excitatory or inhibitory depending on the postsynaptic cell type involved^6^. Signals generated by switching “on” and “off” glutamate-mediated neurotransmission are comprehensively processed within the complex network of retinal cells (bipolar, horizontal, amacrine, and ganglion cells) and sent to optic nerve fibers.

There are two classic types of photoreceptors in mammalian eyes: rods and cones^7^. Although the chemical processes through the visual transduction cascades in these photoreceptors, as described above, are very similar to each other, several functional differences have been experimentally observed. Rods are highly sensitive to light, and visual transduction in rods may be triggered by a single photon. On the other hand, cones require significantly brighter light to activate the signaling cascade and generate electrical signals^8^. Another major difference is reflected in the time course of the photoresponses. The electrical waveforms of the flash light responses are activated and shows slow deactivation, exhibiting a strikingly prolonged response in rods than is observed in cones^8^. The differences in sensitivity and the temporal resolution of the light responses in rods and cones are attributed to different reaction rates throughout the visual phototransduction system. However, the quantitative aspects of the molecular mechanisms underlying distinct phototransduction systems have not yet been conclusively clarified^9^. To quantitatively and systematically investigate how light intensity-dependent photoresponses are divergently regulated in rods and cones, a systems analysis of visual signal transduction systems for these photoreceptors is indispensable.

Former mathematical models of visual transduction cascades for rods and cones, the Hamer 2003^10^ explain fundamental molecular reactions in the phototransduction system. The model includes front-end process of phototransduction, that is, activation process of VP and deactivation of VP* by arrestin, activation of Tr by VP*, activation of PDE by Tr*. The model also includes backend process, that is, cGMP hydrolysis by PDE* and also self-hydrolysis, cGMP production by GC and its inhibition by Ca^2+^, and Ca^2+^ influx by CNG channel current (*I*_CNG_) and efflux by Na^+^/Ca^2+^-K^+^ exchanger. The model well reproduces *I*_CNG_ under single photon stimulation and also for dim-flash regime. The model was improved to Hamer^11^ which was to reproduce *I*_CNG_ under high light level conditions where light adapted characteristics was fairly reproduced by introducing recoverin effect of RK inactivation. More comprehensive netowork model of phototransduction was proposed by Dell’Orco et al.^12^ where the model was based on Hamer 2005 model^11^ but incorporated the effect of RGS9, reformation of Tr, opsin reproduction process and slow activation process by opsin. From these improvements, it could reproduce two-flash characteristics where the VP regeneration network model is necessary.

Their models could well generate macroscopic electrical events actually *I*_CNG_, but do not faithfully reproduce some of the microscopic biochemical responses, e.g., changes in concentrations and activity levels of VP*, Tr, or PDE, probably due to the limited reports for these processes in lower vertebrates, in spite the comprehensive biochemical parameters were thoroughly reported for carp^1,2,8^. If we look at these reports, it is clear that these models cannot reproduce carp biochemical results, for example, the number of phosphorylation of VP is reported as at most 3, while the above models have 7^10^ or 6^12^ phosphorylation sites in its equation. On the other hand, different approach has recently been reported where the possible range of the parameters of the simplified phototransduction model are stochastically estimated from the macroscopic measured current data of CNG channel^13^. This approach is useful when the available information is limited, thus we considered using more rich information related to the phototransduction system to evaluate parameters although the species is not mammalian but carp.

In the present study, frameworks for all the molecular reactions of phototransduction front-end cascades were mathematically elaborated on the basis of the Hamer model^11^ to ensure that a wide variety of light intensity-, ATP-, and GTP-dependent microscopic biochemical reactions are accurately reconstructed.

The proposed carp rod and cone models successfully reproduced in vitro time courses of light intensity-, ATP- and GTP-dependent changes in the concentrations of phosphorylated VPs, Tr*s and PDEs in rods and cones from frog and carp^14,15^. Compared to that of rods, the lower light sensitivity of cones was attributed to the lower affinity of the activated VPs for Trs, as well as the faster desensitization (phosphorylation and inactivation) of VPs. The assumption of an intermediate inactive state, MII^i^, during the thermal decay of activated VP was essential for inducing faster inactivation of VP in rods and possibly also in cones. Furthermore, the combination of faster rates of VP desensitization and RGS9-mediated Tr* inactivation together were indispensable for simulating higher temporal resolution of the electrical waveforms of the light intensity-dependent *I*_CNG_ in vivo experimental systems.

## Methods

A dynamic mathematical model of the visual transduction system in rods and cones was constructed to analyze sets of experimental observations: fundamental microscopic “in vitro” biochemical reactions in phototransduction (Figs. 2, 3, 4).

The fundamental component of the proposed “in vitro” front-end model was developed on the basis of the Hamer model^11^ by elaborating the frameworks for all the key molecular reactions of phototransduction cascades that account for the details of the underlying biochemistry as described in the subsections below. The molecular environment of the membranous disk where all chemical reactions for phototransduction take place in vivo (e.g., concentrations and spatial distributions of membrane-associated molecules, VP, RK, Tr, and PDE, present at the outer segments of photoreceptors), was assumed to be preserved and thus nearly identical to that isolated for biochemical experiments (see Figs. 2, 3, 4). Although the molecular concentrations of in vitro experiments are calculated as shown in the Wet column of Table 1, we assumed that the membrane bound molecular densities are preserved in the in vitro experiments. Taking into account this molecular environment of a single membranous disk, the molecular concentrations of VP, RK, PDE, and Tr were determined from the reported in vivo concentrations^8^ shown in the Sim column of Table 1 (see the “Discussion” section for more details). Note that different concentrations of freely diffusing factors, ATP, GTP, and cGMP, were applied depending on the corresponding biochemical experiments. The simulated results in Figs. 2, 3, 4 were all reproduced using the parameters listed in Table 1 “Sim.” column and Table 3, in the “in vitro” model columns. The results are expressed as events per either VP* or VPtot.

**Table 1:** Parameters of the membrane-associated molecules applied for simulation in comparison to these estimated in the experimental studies.

			VP (µM)	RK (µM)	PDE (µM)	Tr (µM)	ATP (µM)	GTP (µM)
Phosphorylation	Wet	Rod	0.5	0.002	0.0018	0.047	100	500
		Cone	0.5	0.02	0.0018	0.047	100	500
	Sim	Rod	3000	12	11	280	100	500
		Cone	3000	120	11	280	100	500
Tr activation (time-dependent)	Wet	Rod	0.6	0.0024	0.0022	0.056	1000	100
		Cone	0.3	0.012	0.011	0.028	1000	100
	Sim	Rod	3000	12	11	280	1000	100
		Cone	3000	120	11	280	1000	100
Tr activation (light-dependent)	Wet	Rod	3	0.012	0.011	0.28	100	5
		Cone	0.3	0.012	0.0011	0.028	100	5
	Sim	Rod	3000	12	11	280	100	5
		Cone	3000	120	11	280	100	5
PDE activation	Wet	Rod	0.75	0.003	0.0028	0.07	250	100
		Cone	0.75	0.03	0.0028	0.07	250	100
	Sim	Rod	3000	12	11	280	250	100
		Cone	3000	120	11	280	250	100
Photocurrent	Wet	Rod	3000	12	11	280	1000	1000
	Sim	Rod	3000	12	11	280	1000	1000

Wet column represents in vitro substrate concentrations calculated after taking into consideration the experimental test tube volume, while Sim. refers to physiological in vivo concentrations^8^. The Sim. values were applied for the simulation experiments assuming that the in vivo disk membrane structures were preserved in the in vitro experimental environments so the membrane bound molecular concentrations w.r.t. rate constants becomes identical to the in vivo concentrations (see “Discussion” section for more details).

**Figure 1:**
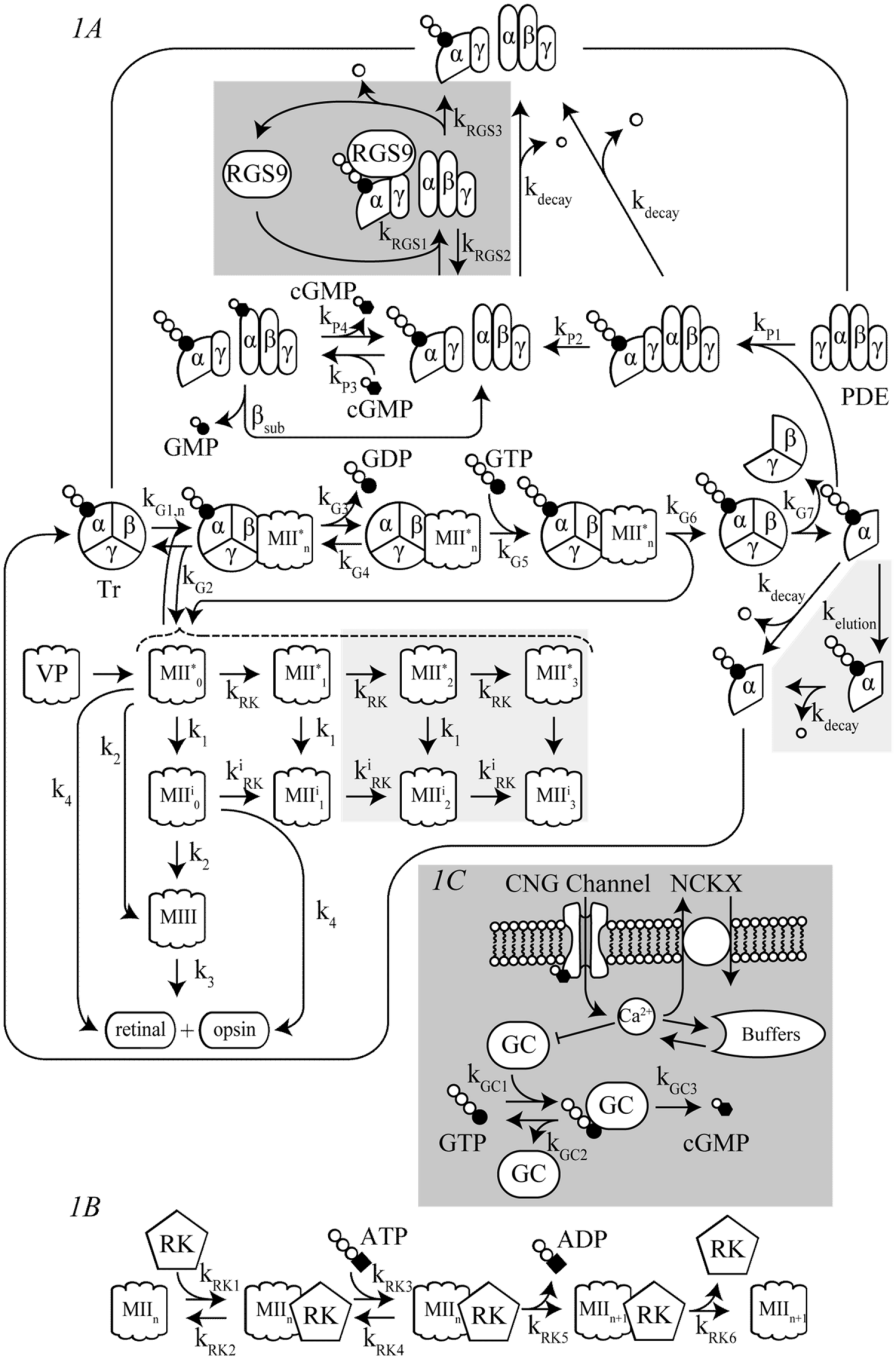
Reaction scheme of visual phototransduction in rods and cones. (**A**) Visual phototransduction, including activation and inactivation of VP, Tr, and PDE, in rods and cones (see Table 2 for abbreviations). (**B**) Details of the phosphorylation reactions. Phosphorylation of VP at 3 sites in vitro (Figs. 2, 3, 4), where only 1 site in vivo (Fig. 5) was assumed. Phosphorylation reactions and Tr* elution indicated in light gray were not included. C, RGS9-dependent inactivation of Tr* and GC-dependent cGMP synthesis, depicted in dark gray, were added for simulating *I*_CNG_ in Fig. 5.

The reaction scheme of the visual transduction cascade is shown in Fig. 1, and the differential equations for the reaction steps in the model are described in the Supplementary materials under Equations. The abbreviations of signaling molecules are summarized in Table 2. The parameters used to define the present model, including the concentrations and binding constants (*K*_*d*_) of signaling factors and the rate constant (*k*), maximum activity (*V*_max_) and half-maximal effective concentration (*K*_1/2_) of substances for activation of enzymes, among other parameters, are listed in Table 3. The initial values of some variables are also listed in Table 4. Model development and simulation-based analyses were both performed with simBio^16^. The time integration of the differential equations was conducted using the Euler method with a time step of 1 µs.

**Table 2:** Abbreviations in model equations.

*k*_RK1_	Rate constant of binding MII_n_* to RK (μM^−1^ s^−1^)
*k*_RK2_	Rate constant of dissociation of MII_n_* from MII_n_*⋅RK_pre_ (s^−1^)
*k*_RK3_	Rate constant of binding MII_n_*⋅RK_pre_ to ATP (μM^−1^ s^−1^_)_
*k*_RK4_	Rate constant of dissociation of MII_n_*⋅RK_pre_ from MII_n_*⋅RK_pre_⋅ATP (s^−1^)
*k*_RK5_	Rate constant of dissociation of ADP from MII_n_*⋅RK_pre_ (s^−1^)
*k*_RK6_	Rate constant of dissociation of MII_n+1_* from MII_n_*⋅RK_post_ following phosphorylation (s^−1^)
*k*^i^_RK1_	Rate constant of binding MII_n_^i^ to RK (μM^−1^ s^−1^)
*k*^i^_RK2_	Rate constant of dissociation of MII_n_^i^ from MII_n_^i^⋅RK_pre_ (s^−1^)
*k*^i^_RK3_	Rate constant of binding MII_n_^i^⋅RK_pre_ to ATP (μM^−1^ s^−1^_)_
*k*^i^_RK4_	Rate constant of dissociation of MII_n_^i^⋅RK_pre_ from MII_n_^i^⋅RK_pre_⋅ATP (s^−1^)
*k*^i^_RK5_	Rate constant of dissociation of ADP from MII_n_^i^⋅RK_pre_ (s^−1^)
*k*^i^_RK6_	Rate constant of dissociation of MII_n+1_^i^ from MII_n_^i^⋅RK_post_ following phosphorylation (s^−1^)
*k*_1_	Rate constant of inactivation of MII_n_* (s^−1^)
*k*_2_	Rate constant of inactivation of MII_n_* and MII_n_^i^ (s^−1^)
*k*_3_	Rate constant of MIII (s^−1^)
*k*_4_	Rate constant of dissociation of retinal and opsin from MII_n_ (s^−1^)
*k*_G1,0_	Rate constant of binding Tr to MII_0_* (μM^−1^ s^−1^)
*k*_G1,1_	Rate constant of binding Tr to MII_1_* (μM^−1^ s^−1^)
*k*_G1,2_	Rate constant of binding Tr to MII_2_* (μM^−1^ s^−1^)
*k*_G1,3_	Rate constant of binding Tr to MII_3_* (μM^−1^ s^−1^)
*k*_G2_	Rate constant of dissociation of MII_n_* from MII_n_*⋅G⋅GDP (s^−1^)
*k*_G3_	Rate constant of dissociation of GDP from MII_n_*⋅G⋅GDP (s^−1^)
*k*_G4_[GDP]	Rate constant of binding GDP to MII_n_*⋅G (s^−1^)
*k*_G5_	Rate constant of binding GTP to MII_n_*⋅G (s^−1^)
*k*_G6_	Rate constant of dissociation of MII_n_* from MII_n_*⋅G⋅GTP (s^−1^)
*k*_G7_	Rate constant of dissociation of Gα⋅GTP from G⋅GTP (s^−1^)
*k*_P1_	Rate constant of binding PDE to Gα⋅GTP (μM^−1^ s^−1^)
*k*_P2_	Rate constant of activation of PDE⋅Gα⋅GTP (s^−1^)
*k*_P3_	Rate constant of binding cGMP to PDE*⋅Gα⋅GTP (μM^−1^ s^−1^)
*k*_P4_	Rate constant of dissociation of cGMP from cG⋅PDE*⋅Gα⋅GTP (s^−1^)
*k*_elution_	Rate constant of elution of Gα⋅GTP (s^−1^)
*k*_RGS1_	Rate constant of binding RGS9 to PDE*⋅Gα⋅GTP (μM^−1^ s^−1^)
*k*_RGS2_	Rate constant of dissociation of RGS9 from RGS9⋅PDE*⋅Gα⋅GTP (s^−1^)
*k*_RGS3_	Rate constant of dissociation of RGS9 from RGS9⋅PDE*⋅Gα⋅GTP and GTP hydrolysis (s^−1^)
*k*_decay_	Rate constant of inactivation of Gα⋅GTP (s^−1^)
*k*_GC1_	Rate constant of binding GC to GTP (s^−1^)
*k*_GC2_	Rate constant of dissociation of cGMP and GC from GC⋅GTP(s^−1^)
*k*_GC3_	Rate constant of dissociation of GMP from GC⋅GTP (s^−1^)
*K*_c_	Calcium ion at which synthesis of cGMP is half of maximum rate of cGMP (μM)
*m*	Hill coefficient for the action of calcium ion on cyclase rate (–)
*β*_dark_	Dark rate of cGMP hydrolysis (s^−1^)
*β*_sub_	Rate constant of cGMP hydrolysis by cG⋅PDE*⋅Gα⋅GTP (s^−1^)
*b*	Ratio of calcium ion to Photocurrent
γ_Ca_	Rate constant of calcium ion extrusion by the NCKX (s^−1^)
*c*_0_	Minimum intracellular calcium ion (μM)
*e*_T_	Concentration calcium ion buffers total (μM)
*k*_b1_	Rate constant of binding calcium ion to buffers (μM^−1^ s^−1^)
*k*_b2_	Rate constant of dissociation of calcium ion from buffers (s^−1^)
*K*_m_	Half maximum of cGMP concentration (μM)
*n*_h_	Hill coefficient for opening CNG channels (–)
*F*	Faraday constant (C/mol)
Vol	Cytoplasmic volume (L)
*G*_max_	Conductance of photocurrent (pA/mV)
*E*_p_	Reversal potential (mV)
*V*_m_	Membrane potential (mV)
VP_total_	Concentration of visual pigment total (μM)
MII*	Concentration of activated metarhodopsin II (μM)
MII^i^	Concentration of intermediate state of activated metarhodopsin II and metarhodopsin III (μM)
RK_free_	Concentration of rhodopsin kinase total (μM)
Tr_free_	Concentration of transducin total (μM)
PDE_free_	Concentration of phosphodiesterase (μM)
RGS9_free_	Concentration of RGS9 free (μM)
cGMP	Concentratino of cGMP (μM)
ATP	Concentration of ATP (μM)
GTP	Concentration of GTP (μM)
GC_free_	Concentration of guanylate cyclase total (μM)
Ca^2+^	Concentration of calcium ion (μM)
Cab	Concentration of intracellular calcium bound to buffers (μM)
*I*_photo_	Photocurrent (pA)

**Table 3:** Parameters in the current models.

Parameter	Unit	In vitro model		Intact model	References	Species
		Rod	Cone	Rod		
*k*_RK1_	μM^−1^ s^−1^	0.462	9.6	0.462	^17^	carp
*k*_RK2_	s^−1^	6.0	80	6.0	^17^	carp
*k*_RK3_	μM^−1^ s^−1^	0.16	0.26	0.16	^17^	carp
*k*_RK4_	s^−1^	1.5	7.0	1.5	^17^	carp
*k*_RK5_	s^−1^	1.083	37.5	1.083	^17^	carp
*k*_RK6_	s^−1^	100	1500	100	^17^	carp
*k*^i^_RK1_	μM^−1^ s^−1^	0.231	4.8	0.231	^17,18^	carp
*k*^i^_RK2_	s^−1^	3.0	40	3.0	^17,18^	carp
*k*^i^_RK3_	μM^−1^ s^−1^	0.08	0.13	0.08	^17,18^	carp
*k*^i^_RK4_	s^−1^	0.75	3.5	0.75	^17,18^	carp
*k*^i^_RK5_	s^−1^	0.542	18.75	0.542	^17,18^	carp
*k*^i^_RK6_	s^−1^	50	750	50	^17,18^	carp
*k*_1_	s^−1^	1.0	120	1.0	^2^	carp
*k*_2_	s^−1^	0.0056	0.0336	0.0056	^2,19^	carp,frog
*k*_3_	s^−1^	0.001	0.006	0.001	^2,19^	carp,frog
*k*_4_	s^−1^	0.0053	0.0318	0.0053	^2,19^	carp,frog
k_G1,0_	μM^−1^ s^−1^	3.57	35.7	3.57	^11,18^	vertebrate, carp
*k*_G1,1_	μM^−1^ s^−1^	1.96	14.5	0	^18^	carp
*k*_G1,2_	μM^−1^ s^−1^	1.08	5.9	0	^18^	carp
*k*_G1,3_	μM^−1^ s^−1^	0.59	2.4	0	^18^	carp
*k*_G2_	s^−1^	50	2250.34	50	^12,18^	vertebrate, carp
*k*_G3_	s^−1^	1000	370.4	1000	^11,18^	vertebrate, carp
*k*_G4_[GDP]	s^−1^	600	2.0	600	^11,18^	vertebrate, carp
*k*_G5_	s^−1^	3.0	1	3.0	^11,18^	vertebrate, carp
*k*_G6_	s^−1^	2000	2000	2000	^11^	vertebrate
*k*_G7_	s^−1^	200	200	200	^11^	vertebrate, carp
*k*_P1_	μM^−1^ s^−1^	250	300	250	^8,20^	carp
*k*_P2_	s^−1^	1000	1000	1000	^8,20^	carp
*k*_P3_	μM^−1^ s^−1^	100	100	100	^8,20^	carp
*k*_P4_	s^−1^	1.0	1.0	1.0	^8,20^	carp
*k*_elution_	s^−1^	80,000	80,000	0.0	^8,20^	carp
*k*_decay_	s^−1^	0.045	0.64	0.045	^10^	carp
VP_total_	μM	3000	3000	3000	^2^	carp
RK_free_	μM	12	120	12	^2^	carp
Tr_free_	μM	280	280	280	^2^	carp
PDE_free_	μM	11	11	11	^2^	carp
ATP	μM	Dependence on experiment	Dependence on experiment	1000	^21–26^	rabbit, frog, bovine
GTP	μM	Dependence on experiment	Dependence on experiment	1000	^21,25^	rabbit, frog

**Table 4:** Initial set of time-dependent variables.

	Rod	Cone
VP_free_	3000	3000
RK_free_	12	120
PDE_free_	11	11
T_free_	280	280
RGS9_free_	3.3	75
GC_free_	8.3	75.9
ATP	1000	1000
GTP_free_	1000	1000
cGMP	2.0	2.0
GC·GTP	4.11	3.90
Ca^2+^	0.5	0.5
Cab	44.4	44.4
*I*_photo_	− 4.0	− 12.0

**Figure 2:**
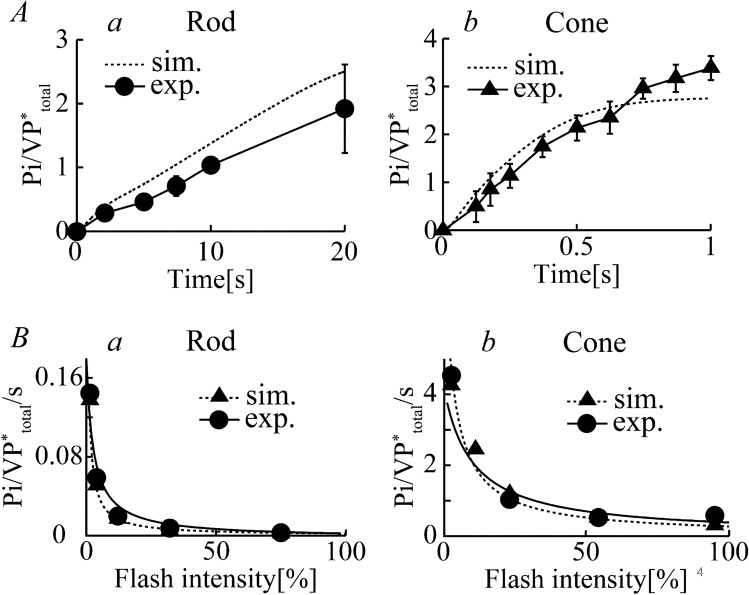
Phosphorylation of visual pigments in rods and cones. (**A**) The time courses for the phosphorylation of VPs (the number of phosphate groups incorporated into an activated visual pigment molecule) measured in the membrane preparations of purified frog rod (a, circle) and carp cone (b, triangle) in response to light flash at 1.3% and 2.5%, respectively, in the experiments^17^. The corresponding simulation results (dotted lines in a and b) are also shown in the figures. (**B**) Maximum rates of phosphorylation reaction per activated visual pigment at different flash intensities in rods (a, circle) and cones (b, circle), determined 10 s and 0.6 s after light stimuli, respectively (data modified from Tachibanaki et al.^17^). Experimental results were fitted by the Michaelis–Menten equation (V/S = Vmax/(S + Km), solid lines). Simulated responses for both the carp rod (a, dotted line triangle) and cone (b, dotted line triangle) were superimposed onto the experimental results.

**Figure 3:**
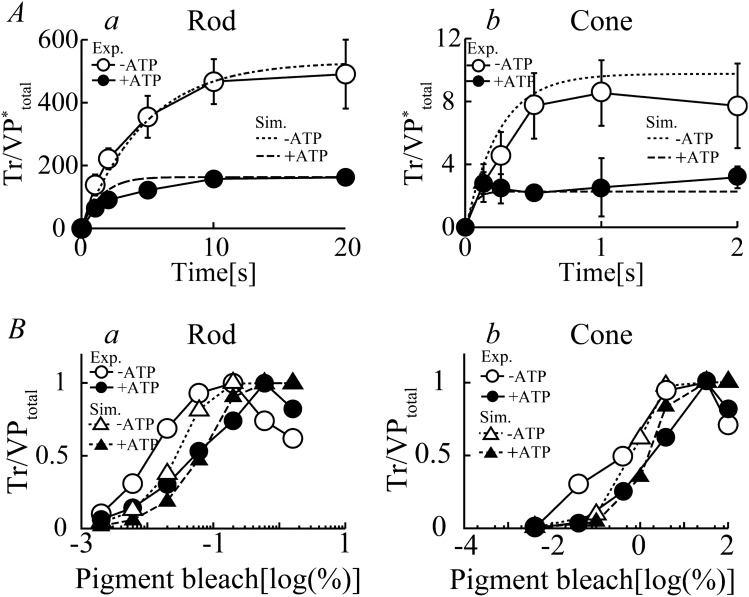
Transducin activation in rods and cones. (**A**) The time courses for Tr activation (the number of GTPγS molecule, a nonhydrolyzable GTP analog, incorporated per VP*) in the membrane preparations of purified carp rods and cones in response to light stimulation (0.0085% for rods (a, circle) and 0.25% for cones (b, circle)) in the presence (filled symbols) and absence (open symbols) of ATP (1 mM) as determined by biochemical experiments^18^. Corresponding simulation results (dotted lines (−ATP) and dashed lines (+ ATP) in a and b were superimposed onto the experimental results. (**B**) Light-induced GTPγS binding as a function of flash intensity in rods (a, circle, after 40 s stimulation) and cones (b, circle, after 20 s stimulation) in the presence (filled symbols) and absence (open symbols) of ATP (0.1 mM) normalized to maximum values. Simulated results for rods (a, triangle) and cones (b, triangle) reproduced under corresponding experimental conditions in the presence (filled symbols) and absence (open symbols) of ATP are also shown in the figure. Values for the rate constants for VP phosphorylation, kRK1–kRK6 and kiRK1–kiRK6, were set to 0 for simulation of experimental results obtained without ATP.

**Figure 4:**
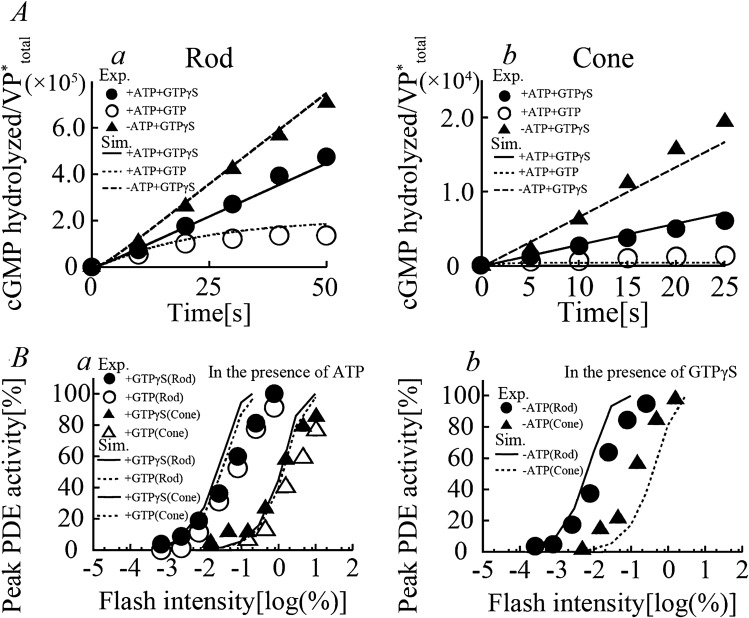
PDE-mediated cGMP hydrolysis in rods and cones. (**A**) The time courses for PDE activities (the number of cGMP molecules hydrolyzed per VP*) in the membrane of carp rods and cones in response to light stimulations (a, 0.024% for rods (open circle); b, 0.46% for cones (open circle)) in the presence of ATP (0.25 mM), cGMP (2500 μM) and either GTP (open closed circle) or GTPγS (filled triangle) determined by biochemical experiments^17,27^). Corresponding simulation results (solid lines (with GTPγS) and dotted lines (with GTP) in a and b) were superimposed onto the experimental data. The continuous presence of saturating levels of cGMP was assumed for the simulation study, and thus βdark was set to 0. (**B**) Light-induced peak PDE activity as a function of flash intensity in rods (circle) and cones (triangle) either in the presence of ATP (a, 0.25 mM) or without ATP (b) and either GTP (open symbols) or GTPγS (filled symbols) are normalized to maximum values. Simulated PDE activity of the rods and cones reproduced under the corresponding experimental conditions (with either GTP (dotted lines) or GTPγS (solid lines)) were superimposed onto the experimental results. The value for the rate constant, *k*_decay_, for cGMP hydrolysis was set to 0 for the simulation of the experimental results obtained with GTPγS.

A wide variety of light intensity-, ATP-, and GTP-dependent photoresponses in distinct types of photoreceptors in carp (extensively described by the biochemical experiments of Kawamura et al.^2,14^, see Figs. 2, 3, 4) were successfully reconstructed and then quantitatively and systematically investigated in the current study.

### Activation of visual pigments

Light stimulation activates a VP, which consists of an opsin combined with the chromophore 11-cis-retinal. The activation of a VP is initiated by light-induced isomerization of 11-cis-retinal to the all-trans form, causing conformational changes in the opsin. The intensity of the light stimulus (*I*_light_), indicated in Figs. 2, 3, 4, are given as a percentage (%), reflecting the % of visual pigments activated (bleached) by the light stimulus directly after the stimulus (at t = 0) when considering the concentration of the total visual pigment as 100% (1).

1 $${I}_{light}=\frac{\left[{VP}^{*}\right]}{\left[{VP}_{total}\right]}$$

Activated VP immediately after light stimulation (MII^*^_0_ at t = 0) is thus determined by (2).

2 $$\left[{MII}_{0}^{*}\right]={I}_{light}\left[{VP}_{total}\right]$$

In the current simulation study, the stimulated VP, MII* (MII^*^_0_–MII^*^_3_, see Fig. 1), is assumed to be capable of activating Tr, while being simultaneously inactivated through two distinct mechanisms: phosphorylation and thermal decay.

### Phosphorylation of visual pigments

The phosphorylation of VPs is mediated by visual pigment-specific kinases (RKs). In the Hamer model, phosphorylation reactions were simply calculated in 3 reaction steps: binding of the activated VP to an RK, phosphorylation of VP, and the dissociation of the RK from the VP. The Hamer model cannot be used to simulate the [ATP]-dependent phosphorylation or sequential inactivation of the VPs. Therefore, the binding of ATP to and dissociation of ADP from the MII*-RK complex were incorporated into the current model (see the reaction scheme in Fig. 1B, reaction formulae (3)–(10) below and the corresponding equations, Eqs. S1–26, S51–60, in the Supplementary materials under Equations) to ensure that the activities of Tr and PDE in the presence and absence of ATP, as estimated by experimental studies, are reconstructed and considered in this study (Figs. 3 and 4)

3 $$MI{I}_{n}^{*}+R{K}_{free} \mathop \rightleftarrows\limits^{{k}_{RK1}}_{{k}_{RK2}} MI{I}_{n}^{*}\cdot R{K}_{pre}$$

4 $$MI{I}_{n}^{*}\cdot R{K}_{pre}+ATP \mathop \rightleftarrows\limits^{{k}_{RK3}}_{{k}_{RK4}} MI{I}_{n}^{*}\cdot R{K}_{pre}\cdot ATP$$

5 $$MI{I}_{n}^{*}\cdot R{K}_{pre}\cdot ATP \mathop \rightarrow\limits^{{k}_{RK5}} MI{I}_{n+1}^{*}\cdot R{K}_{post}+ADP$$

6 $$MI{I}_{n+1}^{*}\cdot R{K}_{post} \mathop \rightarrow\limits^{{k}_{RK6}} MI{I}_{n+1}^{*}+R{K}_{free}$$

7 $$MI{I}_{n}^{i}+R{K}_{free} \mathop \rightleftarrows\limits^{{k}_{RK1}^{i}}_{{k}_{RK2}^{i}} MI{I}_{n}^{i}\cdot R{K}_{pre}$$

8 $$MI{I}_{n}^{i}\cdot R{K}_{pre}+ATP \mathop \rightleftarrows\limits^{{k}_{RK3}^{i}}_{{k}_{RK4}^{i}} MI{I}_{n}^{i}\cdot R{K}_{pre}\cdot ATP$$

9 $$MI{I}_{n}^{i}\cdot R{K}_{pre}\cdot ATP \mathop \rightarrow\limits^{{k}_{RK5}^{i}} MI{I}_{n+1}^{i}\cdot R{K}_{post}+ADP$$

10 $$MI{I}_{n+1}^{i}\cdot R{K}_{post} \mathop \rightarrow\limits^{{k}_{RK6}^{i}} MI{I}_{n+1}^{i}+R{K}_{free}$$

where $0\le \mathrm{n}\le 3$ (n = number of phosphorylated VP sites) when simulating photoresponses in vitro (Figs. 2, 3, 4). The notation “pre” and “post” in above reaction distinguish RK-bound states of R* before and after phosphorylation, respectively.

Tachibanaki et al.^17^ showed that the steady-state phosphorylation level in cones was ~ 3, which is in good agreement with the number of phosphates necessary for the complete suppression of the activated VPs in rods^28^. Although a maximum of 9 phosphorylation sites in the C terminus of MII have been suggested^29^, only 3 sites were considered in the current model (for Figs. 2, 3, 4, $0\le \mathrm{n}\le 2$ for (3)–(10)) based on biochemical experiment observations in vitro^17,28^. For the calculation of the visual pigment phosphorylation shown in Fig. 2, the total number of phosphate groups incorporated into activated visual pigments was determined by Eq. (11).

11 $$\begin{aligned} \left[ {{\text{Pi}}} \right] &= \sum\limits_{{{\text{n}} = 1}}^{2} {\left\{ {\left( {\left[ {{\text{MII}}_{{\text{n}}}^{*} \cdot {\text{RK}}_{{{\text{pre}}}} } \right] + \left[ {{\text{MII}}_{{\text{n}}}^{*} \cdot {\text{RK}}_{{{\text{pre}}}} \cdot {\text{ATP}}} \right] + \left[ {{\text{MII}}_{{\text{n}}}^{{\text{i}}} \cdot {\text{RK}}_{{{\text{pre}}}} } \right] + \left[ {{\text{MII}}_{{\text{n}}}^{{\text{i}}} \cdot {\text{RK}}_{{{\text{pre}}}} \cdot {\text{ATP}}} \right]} \right) \times {\text{n}}} \right\}} \hfill \\ & \quad+ \sum\limits_{{{\text{n}} = 1}}^{3} {\left\{ {\left( {\left[ {{\text{MII}}_{{\text{n}}}^{*} } \right] + \left[ {{\text{MII}}_{{\text{n}}}^{*} \cdot {\text{RK}}_{{{\text{post}}}} } \right] + \left[ {{\text{MII}}_{{\text{n}}}^{{\text{i}}} } \right] + \left[ {{\text{MII}}_{{\text{n}}}^{{\text{i}}} \cdot {\text{RK}}_{{{\text{post}}}} } \right]} \right) \times {\text{n}}} \right\}} \hfill \\ \end{aligned}$$

Rate constants for the phosphorylation reactions of the VPs, *k*_RK1_–*k*_RK6_, were estimated by manually fitting to the experimental data exhibiting the time courses and the maximal rate of phosphorylation in rod and cone at given light flash intensities^17^ (see Fig. 2) The reaction rates for the phosphorylation of the activated VPs in the Hamer model were designed to vary as the phosphorylation reactions proceed, whereas those for the current model were set as constant parameters since the rate of phosphorylation up to 3P_i_/VP^*^_total_ does not seem to vary significantly (see Fig. 1 in Tachibanaki et al.^17^). The phosphorylation rate constants for MII^i^, *k*^i^_RK1_–*k*^i^_RK6_, were assumed to be one-half those of MII* (*k*_RK1_–*k*_RK6_) to reproduce continuing phosphorylation processes even after VP activity was completely terminated (based on the comparison of the time course of VP phosphorylation (see Fig. 2A) with that of Tr activation in the presence of ATP (Fig. 3A)^17,18^.

### Thermal decay of visual pigments

Thermal decay of VPs is another pathway for the inactivation mechanism. The process is mediated by conformational changes in which MII^*^_0_ transitions to MIII^30^ directly or after undergoing a transition to an intermediate state, MII^i^^31^ (see the “Discussion” section), as well as by bleaching (dissociation of retinal from opsin) and the subsequent recycling of VPs^27^. MIII may also undergo bleaching processes (see the reaction scheme in Fig. 3, reaction formulae (12)–(17) below, and corresponding equations, Eqs. S1–4, S14–17, S27–28, S61–62, in the Supplementary materials under Equations).

12 $$MI{I}_{n}^{*}\stackrel{\hspace{1em}{k}_{1}\hspace{1em}}{\to }MI{I}_{n}^{i} \left(n\ge 0\right)$$

13 $$MI{I}_{0}^{*}\stackrel{\hspace{1em}{k}_{2}\hspace{1em}}{\to }MIII$$

14 $$MI{I}_{0}^{i}\stackrel{\hspace{1em}{k}_{2}\hspace{1em}}{\to }MIII$$

15 $$MIII\stackrel{\hspace{1em}{k}_{3}\hspace{1em}}{\to }retinal+opsin$$

16 $$MI{I}_{0}^{*}\stackrel{\hspace{1em}{k}_{4}\hspace{1em}}{\to }retinal+opsin$$

17 $$MI{I}_{0}^{i}\stackrel{\hspace{1em}{k}_{4}\hspace{1em}}{\to }retinal+opsin$$

Rate constants for the inactivation reaction of the VPs, *k*_2_–*k*_4_, were determined based on the reports of Kolesnikov et al.^19^ and Kawamura et al.^2^, while *k*_1_ was estimated manually by model fitting. Note that the rates of *k*_2_–*k*_4_ were assumed to be sixfold faster, while *k*_1_ was 120-fold faster in cones than in rods, since the lifetime of the MII intermediate was expected to be at least tenfold shorter in cones by referring to –ATP conditions in Fig. 3Aa and b. The reaction rates for inactivation were verified by reproducing Tr activation in the absence of ATP under the condition that no VP phosphorylation occurs, as shown in Fig. 3.

### Activation and inactivation of transducins

Transducin (Tr) is a prototypic heterotrimeric G protein comprising G_α_ and G_βγ_ subunits. Stimulated visual pigments increase the catalytic activity of Tr by exchanging GTP for the GDP previously bound to G_α_. G_α_-GTP then dissociates from the G_βγ_ subunits and subsequently binds to a PDE, increasing the degradation rate of cGMP (see the reaction scheme in Fig. 3, reaction formulae (18)–(22) below, and corresponding equations, Eqs. S29–42, S63–67, in the Supplementary materials under Equations). In the experiment, the number of GTPγS, a nonhydrolyzable GTP analog, incorporated per Tr* (G_α_-GTPγS) was measured as an indicator of activated Tr.

18 $$MI{I}_{n}^{*}+G\cdot GDP \mathop \rightleftarrows\limits^{{k}_{G1n}}_{{k}_{G2}} MI{I}_{n}^{*}\cdot G\cdot GDP$$

19 $$MI{I}_{n}^{*}\cdot G\cdot GDP \mathop \rightleftarrows\limits^{{k}_{G3}}_{{k}_{G4}} MI{I}_{n}^{*}\cdot G+GDP$$

20 $$MI{I}_{n}^{*}\cdot G+GTP\stackrel{\hspace{1em}{k}_{G5}\hspace{1em}}{\to }MI{I}_{n}^{*}\cdot G\cdot GTP$$

21 $$MI{I}_{n}^{*}\cdot G\cdot GTP\stackrel{\hspace{1em}{k}_{G6}\hspace{1em}}{\to }MI{I}_{n}^{*}+G\cdot GTP$$

22 $$G\cdot GTP\stackrel{\hspace{1em}{k}_{G7}\hspace{1em}}{\to }{G}_{\alpha }\cdot GTP+{G}_{\beta \gamma }$$

where *n* indicates the number of phosphorylated sites (*n* ≥ 0).

For the current model, the constraint of mass conservation was newly introduced to the concentration of Tr to prevent the continuous increase in G_α_-GTPγS in response to a stronger light stimulus (see Fig. 3B). Since GTPγS is not hydrolyzable, the initial rate of the change in G_α_-GTPγS (Fig. 3A) purely reflects the rate of Tr activation because the VP* desensitization (inactivation and phosphorylation) reactions progress more slowly than those of Tr activation^32^. The rate constants for the reactions of Tr activation, *k*_G1,n_–*k*_G7_, were thus estimated by manually model fitting to the initial rate of Tr activation in response to light flash stimulation (0.0085% for rods and 0.25% for cones) as well as the light intensity-dependent activation of Tr in vitro^18,32^ (see Fig. 3). Note that the affinity of G_α_-GTP for MII^*^_0_ (*k*_G1,0_/*k*_G2_) was approximately sixfold higher in rods than in cones. The estimation was comparable to that of Chen et al.^33^.

The G_α_-GTP-binding rate to MII^*^_1_ (*k*_G1,1_) was estimated to be ~ 60% of that to MII^*^_0_ (*k*_G1,0_), while the rate (*k*_G1,2_ and *k*_G1,3,_ see (23)) was assumed to further decrease through successive phosphorylation of the VPs (to MII^*^_2_ and MII^*^_3_, respectively), based on Gibson et al.^34^ (see the “Discussion” section for more details).

23 $${k}_{G1,n}={k}_{G10}\mathit{exp}(-\omega n)$$

The reaction rates for VP inactivation were verified by reproducing the experimental data obtained in the absence of ATP (Fig. 3) under the condition that VP is not phosphorylated. The values of *k*_G1,n_–*k*_G7_ determined in the present study were comparable to these of the Hamer model^11^.

Physiologically, activated Tr^*^ (G_α_-GTP) undergoes inactivation by the hydrolysis of its bound GTP to GDP on a minute time scale^18^ (see the reaction scheme in Fig. 1, reaction formulae (24)–(26) below, and corresponding equations, Eqs. S42–45, S67–69, in the Supplementary materials under Equations). Calculating the elution of Tr* from the localized outer membrane complex where phototransduction takes place in experimental systems in vitro was indispensable for the reconstruction of the decay of PDE activity (Fig. 4). Notably, inactivation does not occur with GTPγS.

24 $$PD{E}^{*}\cdot{G}_{\alpha }\cdot GTP \mathop \rightarrow\limits^{{k}_{decay}} PDE+{G}_{\alpha }\cdot GDP+Pi$$

25 $$PDE\cdot{G}_{\alpha }\cdot GTP \mathop \rightarrow\limits^{{k}_{decay}} PDE+{G}_{\alpha }\cdot GDP+Pi$$

26 $${G}_{\alpha }\cdot GTP \mathop \rightarrow\limits^{{k}_{decay}} {G}_{\alpha }\cdot GDP+Pi$$

### Regulation of PDE activity

**Figure 5:**
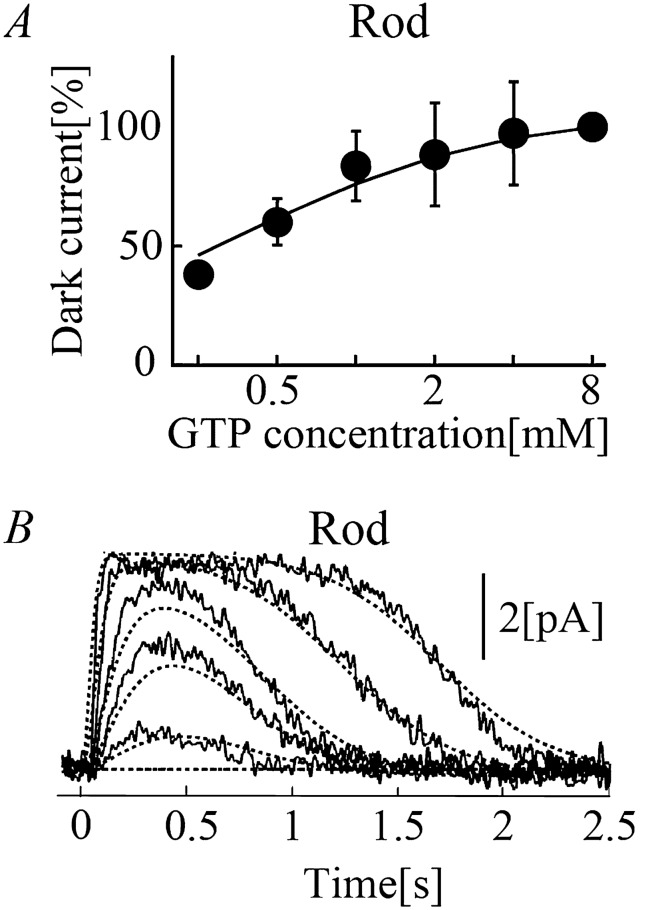
*I*_CNG_ in rods and cones. (**A**) [GTP]_i_-dependent *I*_CNG_ recorded from a truncated outer segment of frog rods (circle), normalized to *I*_CNG_ at 8 mM [GTP]_i_^14^. Corresponding simulation results at steady-state were superimposed onto the experimental data (solid line). (**B**) Time courses of light-induced *I*_CNG_ as a function of flash intensity recorded from the outer segments of the carp rods (light stimuli; 5.1E−6, 1.6E−5, 5.1E−5, 1.6E−4, and 5.1E−4%)^14^. Simulated *I*_CNG_ for rods reproduced under the corresponding experimental conditions was superimposed onto the experimental results (dotted lines).

The catalytic activity of PDE in hydrolyzing cGMP at rest is elevated when the inhibitory γ subunit is removed from the enzyme upon binding activated Tr [PDE^*^⋅G_α_-GTP, see (27–32)]. The activity of PDE thus decreases as G_α_-GTP is hydrolyzed by its GTPase activity. For the Hamer model, cGMP hydrolysis by PDE was simply described by a rate constant^11^. In contrast, in the current study, the chemical reaction of cGMP binding to PDE was calculated to indicate that cGMP hydrolysis (30) is also a [cGMP]_i_-dependent process (see the reaction scheme in Fig. 1, reaction formulae (27)–(30) below, and corresponding equations, Eqs. S42, S44–S45 and S67–S70, in the Supplementary materials under Equations). Notably, the PDE-independent basal degradation of cGMP was also assumed when the calculating steady-state [cGMP]_i_ in the dark, taking into account the continuous cGMP generation by guanylate cyclase (GC) in vivo (see Fig. 5).

27 $$PDE+{G}_{\alpha }\cdot GTP\stackrel{\hspace{1em}{k}_{P1}\hspace{1em}}{\to }PDE\cdot {G}_{\alpha }\cdot GTP$$

28 $$PDE\cdot {G}_{\alpha }\cdot GTP\stackrel{\hspace{1em}{k}_{P2}\hspace{1em}}{\to }PD{E}^{*}\cdot {G}_{\alpha }\cdot GTP$$

29 $$PD{E}^{*}\cdot {G}_{\alpha }\cdot GTP+cGMP \mathop \rightleftarrows\limits^{{k}_{P3}}_{{k}_{P4}} cG\cdot PD{E}^{*}\cdot {G}_{\alpha }\cdot GTP$$

30 $$cG\cdot PD{E}^{*}\cdot {G}_{\alpha }\cdot GTP \mathop \rightarrow\limits^{{\beta }_{sub}} PD{E}^{*}\cdot {G}_{\alpha }\cdot GTP+GMP$$

Based on Kawamura et al.^8^, G_α_-GTP was also assumed to be eluted from the localized outer membrane complex where phototransduction takes place in the in vitro system (see (31) and the “Discussion” section). The eluted G_α_-GTP was also presumed to be inactivated, as described in the previous section (see (32)).

31 $${G}_{\alpha }\cdot GTP \mathop \rightarrow\limits^{{k}_{elution}} {G}_{\alpha }\cdot GT{P}_{eluted}$$

32 $${G}_{\alpha }\cdot GT{P}_{eluted} \mathop \rightarrow\limits^{{k}_{decay}} {G}_{\alpha }\cdot GDP+Pi$$

The rate constants for the reactions of PDE activation (*k*_P1_ and *k*_P2_), cGMP hydrolysis (*k*_P3_, *k*_P4_, and *β*_sub_) and Tr* (G_α_-GTP) inactivation (*k*_decay_) were estimated manually by model fitting to the time courses of cGMP hydrolysis per VP* in response to a given light flash stimulation and to light intensity-dependent PDE activity^2,8,20^ (see Fig. 4).

### Simple “intact” system for validating the phototransduction system with *I*_CNG_

Although the above proposed front-end phototransduction system can reproduce the in vitro experimental results, the system must be compatible with backend photocurrent (*I*_CNG_) generating system. However, since the system becomes too complex, thus the model parameters becomes difficult to validate with experimental data. Thus, in this paper, we confirmed that the proposed phototransduction system can generate reasonable *I*_CNG_ at least with one set of parameters for the simplified photocurrent generating system which was based on the Hamer model^10^ and Dell’Orco model^12^.

For the calculations of the light intensity-dependent macroscopic photoresponses observed in “intact” retinal photoreceptor cells (Fig. 5), the effects of cytosolic factors, e.g., arrestin (Arr) and G protein signaling 9 (RGS9), were considered in addition to the “in vitro” system. The *I*_CNG_ presented in Fig. 5 were calculated using the parameters listed in Table 5, in the “intact” model columns, and as described in detail in each subsection below.

**Table 5:** Parameters in the intact models.

Parameter	Unit	In vitro model		Intact model	References	Species
		Rod	Cone	Rod		
*k*_RGS1_	μM^−1^ s^−1^	–	–	0.85	^8^	carp
*k*_RGS2_	s^−1^	–	–	0.05	^8^	carp
*k*_RGS3_	s^−1^	–	–	5.0	^8^	carp
*k*_GC1_	s^−1^	0.1	0.006	0.1	^14,35^	frog, carp
*k*_GC2_	s^−1^	100	108.45	100	^14,35^	frog, carp
*k*_GC3_	s^−1^	8.5	8.967	8.5	^14,35^	frog, carp
*K*_c_	μM	–	–	0.25	^36^	bovine
*m*	–	–	–	2.0	^11^	vertebrate
*β*_dark_	s^−1^	–	–	3.5	^9^	carp
*β*_sub_	s^−1^	2000	2400	2000	^2^	carp
*b*	μMs^−1^pA^−1^	–	–	0.303	^8,37^	carp, salamander, bass
γ_Ca_	s^−1^	–	–	106.7	^8^	carp
*c*_0_	μM	–	–	0.01	^12^	vertebrate
*e*_T_	μM	–	–	400	^12^	vertebrate
*k*_b1_	μM^−1^ s^−1^	–	–	4.0	^8^	carp
*k*_b2_	s^−1^	–	–	16	^8^	carp
*K*_m_	μM	–	–	20	^8,35^	carp
*n*_h_	–	–	–	2.0	^8,35^	carp
F	C/mol	–	–	96,486.7		
vol	L	–	–	1.2e−13	^8^	carp
G_max_	pA/mV	–	–	10.1	^8^	carp
*K*_p_	mV^−1^	–	–	0.03	^8^	carp
*E*_p_	mV	–	–	10	^38^	lizard
*V*_m_	mV	–	–	− 30	^38^	lizard
RGS9_free_	μM	–	–	3.3	^2^	carp
cGMP	μM	–	–	2.0	^39^	triturus
GC_free_	μM	4.2	72	4.2	^35^	carp
Ca^2+^	μM	–	–	0.5	^11^	vertebrate
Cab	μM	–	–	44.4	^8^	carp
*I*_photo_	pA	–	–	− 4	^2^	carp

For the simulation of the electrical waveforms of the light intensity-dependent photoresponses shown in Fig. 5B, the phosphorylation reaction was calculated only for a single site [*n* = 0 for (3–10)], assuming a complete loss of VP activity quickly after arrestin (Arr) binds to MII^*^_1_ in the “intact” system. In this case, MII^*^_0_ was assumed to be only capable of activating Tr (see the “Discussion” section).

The elution of G_α_-GTP was estimated to be negligible in the “intact” system when simulating the electrical waveforms of the light intensity-dependent photoresponses in Fig. 5.

### Increase in PDE activation of transducin by RGS9

The GTPase activity of PDE-associated G_α_ dramatically increases as the PDE⋅G_α_GTP complex binds to Regulator of RGS9, a GTPase-accelerating protein (see the reaction formulae (33), (34) below and corresponding equations, Eqs. S69 and 71, in the Supplementary materials under Equations). The RGS9 effects on free Tr* (G_α_-GTP) were assumed to be negligible since the binding affinity of RGS9 for free Tr* was considerably lower than it was for PDE-bound Tr*^40^

33 $$PD{E}^{*}\cdot{G}_{\alpha }\cdot GTP+RGS9 \mathop \rightleftarrows\limits^{{k}_{RGS1}}_{{k}_{RGS2}} RGS9\cdot PD{E}^{*}\cdot{G}_{\alpha }\cdot GTP$$

34 $$RGS9\cdot PD{E}^{*}\cdot{G}_{\alpha }\cdot GTP \mathop \rightarrow\limits^{{k}_{RGS3}} PDE+{G}_{\alpha }\cdot GDP+Pi+RGS9$$

The rate constants for RGS9-dependent hydrolysis, *k*_RGS1_–*k*_RGS3_, were estimated by model fitting to the time courses of the electrical waveforms of the light intensity-dependent photoresponses, which are shown in Fig. 5 recorded in a physiological condition^8^ (see the “Discussion” section). Based on Tachibanaki et al.^18^, an approximate 20-fold higher expression of RGS9 in cones than in rods was assumed for the simulation (see Table 3). The effect of RGS9 was excluded from the current model for the simulation of the in vitro experiments (Figs. 2, 3, 4) under the assumption that RGS9 is translocated from the membranous disk in the outer segments to the inner segments of photoreceptors during sample preparation^41^ (see the “Discussion” for more details).

### Regulation of guanylate cyclase (GC)

Physiologically, the concentration of intracellular cGMP is determined by the balance between the rate of cGMP production and degradation. The rate of GTP-dependent cGMP production by GC was negatively regulated by Ca^2+^ (see the reaction scheme in Fig. 1, reaction formula (36) below, and corresponding differential equation, Eq. S72, in the Supplementary materials under Equations).

35 $$G{C}_{free}+GTP\begin{array}{c}\stackrel{\hspace{1em}{k}_{GC1}\hspace{1em}}{\to }\\ \underset{\hspace{1em}{k}_{GC2}\hspace{1em}}{\leftarrow }\end{array}GC\cdot GTP$$

36 $$GC\cdot GTP\stackrel{\hspace{1em}\frac{{k}_{GC3}}{1+(\frac{[C{a}^{2+}]}{Kc}{)}^{m}}\hspace{1em}}{\to }G{C}_{free}+cGMP+2Pi$$

The half-maximal value of the cytosolic Ca^2+^ concentration ([Ca^2+^]_i_) for the inhibition of GC (*K*c) and the Hill coefficient for Ca^2+^-dependent inhibition were determined based on experimental reports^42–45^. The rate constants for cGMP production, *k*_GC1_–*k*_GC3_, and for basal cGMP degradation, *β*_dark_, were estimated by adjusting the resting cGMP level at 2 μM^46^ given that [Ca^2+^]_i_ in the dark is ~ 500 µM^47–49^. The values of *k*_GC1_–*k*_GC3_ and *β*_dark_ determined in the present study were comparable to those estimated by Kawamura et al.^8^ and other experimental studies^10,11,50,51^.

### Regulation of CNG currents in the “intact” system

[cGMP]_I_ regulates CNG currents (*I*_CNG_) in the outer segments of photoreceptor cells (see the corresponding differential equation, Eq. S83, in the Appendix under Equations). The half-maximal value of [cGMP]_i_ for the activation of *I*_CNG_ (*K*_m_) and the Hill coefficient for its [cGMP]_i_-dependent activity (n_h_) was determined based on experimental reports^35,52–54^. The maximum conductance of *I*_CNG_, *G*_max_, in the dark was estimated by reproducing the steady-state current of ~ 4 pA in the rods, given that membrane potential (V_m_) of each photoreceptor cells under the scotopic conditions is ~ 40 mV^55,56^.

### Regulation of [Ca^2+^]_i_ in the “intact” system

[Ca^2+^]_I_ in the current model is regulated by Ca^2+^ influx through *I*_CNG_, Ca^2+^ efflux via Na^+^/Ca^2+^-K^+^ exchangers (NCKX), and Ca^2+^ binding to endogenous calcium buffer (see the reaction scheme in Fig. 1C and the corresponding differential equations in the Appendix under Equations: for free Ca^2+^ ([Ca^2+^]_i_), Eq. S81, and for bound Ca^2+^ (Cab), Eq. S82).

The relative Ca^2+^ permeability of *I*_CNG_ (*b*) was set at 0.3^57^. The minimum Ca^2+^ concentration within cells (*c*_0_) was set at 0.01 μM based on experimental reports^11,47,49^. The conductance of the NCKX (γ_Ca_), total content of endogenous Ca^2+^ buffer (*e*_T_) and the rate constants for Ca^2+^ binding to the endogenous buffer (*k*_b1_ and *k*_b2_) were estimated by reproducing the resting [Ca^2+^]_i_ at 500 nM under scotopic conditions, considering that [Ca^2+^]_i_ may be reduced to ~ 100 nM when *I*_CNG_ decrease to 0 pA in response to a strong light stimulus. The values of γ_Ca_ determined in the present study were comparable to those estimated by Hamer et al.^10^.

## Results

### Phosphorylation of visual pigments

Activated VPs undergo processes of inactivation mediated by visual pigment-specific kinases (RKs). Time courses of visual pigment phosphorylation in membrane preparations of rods and cones in response to given light flash stimulation (Fig. 2A, 1.3% for rods (*a*, circle) and 2.5% for cones (*b*, triangle)) were reported by Tachibanaki et al.^17^. VP phosphorylation in cones was evidently faster than it was in rods (half-maximum phosphorylation: ~ 12.5 s in rods and ~ 250 ms in cones), whereas the rate of phosphorylation depended on the flash intensity (see below). The in vitro elements of the current visual transduction cascade model well reproduced the time course of phosphorylation of visual pigments in both rods and cones (see dotted lines). The simulation study clarified that the difference in the apparent rates of phosphorylation in these two types of photoreceptors was due to distinct amounts of receptor kinases (12 µM in rods and 120 µM cones) and reaction rates for each chemical process during the phosphorylation of the VPs in the rods and cones (*k*_RK1_–*k*_RK6_ and *k*^i^_RK1_–*k*^i^_RK6,_ see Table 3), as predicted by Tachibanaki et al.^17^.

Tachibanaki and colleagues have also reported distinct maximum rates of phosphorylation at different flash intensities (Fig. 2B) in membrane preparations of rods (*a*, circle) and cones (*b*, circle)^17^. Fitting the experimental results by the Michaelis–Menten equation (*V*/*S* = *V*_max_/(*S* + *K*_*m*_), where S = VP*/VP_tot_ (expressed as a percentage)) yielded an estimate of *V*_max_ (the maximum phosphorylation rates) and *K*_m_ (the half-maximal values of VP*/VP_tot_ upon stimulation of the RKs) for the phosphorylation reactions of 0.0049 Pi/VP*/s (or 0.037 pmol Pi/s) and 0.61% in rods and 0.41 Pi/VP*/s (or 3.1 pmol Pi/s) and 10% in cones, respectively. *V*_*max*_ and *K*_m_ were determined by fitting the same equation to the corresponding simulation results for rods (Fig. 2Ba, triangle) and cones (Fig. 2Bb, triangle), and comparable values were obtained (0.0018 Pi/VP*/s and 0.36% in rods; 0.32 Pi/VP*/s and 4.8% in cones, respectively), indicating that the current model well reconstructed the flash intensity-dependent phosphorylation of VPs in rods and cones. These results showed that, with the chosen rate constants for the phosphorylation reaction of the VPs (*k*_RK1_–*k*_RK6_ and *k*^i^_RK1_–*k*^i^_RK6_) in both photoreceptors, the model provided reasonable descriptions of the experimental data.

### Activation of Tr (G_α_-GTP)

Stimulated visual pigment increases the catalytic activity of Tr in exchanging GTP for GDP. The time courses of Tr activation in the membrane preparations of rods and cones in response to given light stimulation (Fig. 3A, 0.0085% for rods (*a*, circle) and 0.25% for cones (*b*, triangle)), in the presence (filled symbols) and absence of ATP (open symbols) were reported by Tachibanaki et al.^18^. For these experiments, the number of GTPγS molecules, a nonhydrolyzable GTP analog, incorporated per VP* was measured as an indicator of activated Tr. The time course of GTPγS binding to Tr in the absence of ATP, thus without VP phosphorylation, was fitted with a simple exponential function ($Y=A[1-\mathrm{exp}\left(-kt\right)]$). The estimated initial rates (*A*k) of Tr activation were 143 and 30 Tr*/VP*/s for rods and cones, respectively, suggesting that amplification of the incoming light signal at the level of Tr activation was approximately fivefold more efficient in rods than it was in cones. Even in the absence of ATP, GTPγS-binding reactions (A) led to eventual saturation (~ 497 GTPγS/VP* in rods and ~ 8.5 GTPγS/VP* in cones) due to termination of MII*_0_ activity because of inactivation processes. In the presence of ATP (1 mM), the maximum amount of GTPγS bound to Tr per VP* was diminished by ~ 60% in the membrane preparations of both types of photoreceptor cells owing to the faster desensitization of MII* by the additional phosphorylation processes. The in vitro elements of the current model well reconstructed time courses of Tr activation for both rods and cones in the presence and absence of ATP (Fig. 3A, dashed lines (+ATP) and dotted lines (−ATP)) when corresponding light stimulation intensities (*a*, 0.0085% in rods, *b*, 0.25% in cones) were applied. As Tachibanaki et al.^32^ suggested, the initial rates of Tr activation with ATP (~ 52.7 Tr*/VP*/s in rods and ~ 15.8 Tr*/VP*/s in cones), were almost the same to those in the absence of ATP for both types of photoreceptors (~ 52.7 Tr*/VP*/s in rods and ~ 15.8 Tr*/VP*/s in cones), indicating that the molecular reactions involved in VP* phosphorylation progressed relatively slowly compared to those of Tr activation.

Tachibanaki and colleagues have also reported light-induced Tr activation at different flash intensities (Fig. 3B) in membrane preparations of rods (*a*, circle) and cones (*b*, circle) in the presence (filled symbols) and absence (open symbols) of ATP (100 μM)^32^. The half-maximal flash intensity for the activation of Tr was approximately 100-fold higher in cones than it was in rods. It was also evident that ATP reduced the light sensitivity of Tr activation in both rods and cones, reflecting the facilitated desensitization of MII*_0_ through phosphorylation processes. The model also well simulated light intensity-dependent Tr activation in both rods and cones. Thus, with the rate constants for the Tr activation reactions (*k*_G1,0_–*k*_G1,3_ and *k*_G2_–*k*_G6_) and the reaction rates for the VP phosphorylation and inactivation processes with and without ATP in both types of photoreceptors, the model provided reasonable descriptions of the experimental data.

As mentioned above, hypothetical intermediate state MII^i^ was essential to reproduce transducin activation results. MII which is active state of VP, is deactivated by the three process in the in vivo environment, (1) phosphorylation process to MII_n_ ($\mathrm{n }\ge 1$), (2) arrestin binding which may occur for phosphorylated MII, and (3) self-deactivation where transition to MIII is reported. In the in vitro condition of wet experiment of transducin activation with no ATP shown in Fig. 3A –ATP condition (open circle in Fig. 3Aa and b), arrestin does not exist since it is washed out in the membrane preparation, and phosphorylation does not occur since there is no ATP in the solution. Thus the termination of transducin activation was considered to be due to the self-deactivation process of MII. However, the transition rate of MII to MIII (*k*_2_) is reported to be very slow^2,19^, thus we assumed intermediate inactive state MII^i^ which is not MIII but has no transducin activation ability.

### Hydrolysis of cGMP by PDE

The catalytic activity of PDE in hydrolyzing cGMP increases when the gamma inhibitory subunit is removed from the enzyme upon binding Tr* (G_α_-GTP). The PDE activity thus decreases as G_α_-GTP is hydrolyzed by its GTPase activity. Koshitani et al.^20^ reported time courses of cGMP hydrolysis in membrane preparations of rods and cones in response to light stimulations (Fig. 4A, 0.024% for rods (*a*, open circle) and 0.46% for cones (*b*, open circle)) in the presence and absence of ATP (solid and dashed lines, respectively) with GTPγS (filled symbols) and GTP (open symbols). In rods, the number of cGMP hydrolyzed per activated VP* monotonically increased with GTPγS increases, in either the presence or absence of ATP. On the other hand, cGMP hydrolyzed with ATP and GTP reached saturation at ~ 1/3 of that with GTPγS at 50 s after the light stimulus due to termination of PDE activity as GTP hydrolysis preceded. In contrast, cGMP hydrolysis measured with ATP and GTPγS in cones was diminished by ~ 99% relative to that in rods and was further reduced to ~ 1/6 with GTP, 25 s after the light stimulus. The lifetime of Tr was thus estimated to be ~ twofold shorter in cones^20^.

Peak PDE activities (first derivatives of the number of cGMPs hydrolyzed per activated VP* molecule) were also measured at various intensities of light stimulation (Fig. 4Ba, circle for rods and triangle for cones) in the presence of ATP and either GTP (open symbols) or GTPγS (filled symbols). The peak light intensity-dependent PDE activity with GTP (open symbols) and GTPγS (filled symbols) was not drastically different in rods and cones, indicating that the rate of GTP hydrolysis is slower than that of GTP-dependent or GTPγS-dependent activation of PDE. However, the peak PDE activity was significantly more sensitive to light when the phosphorylation of VPs was prohibited, that is, when ATP was absent (the comparison is shown in Fig. 4Bb to a).

The in vitro elements of the current model well reconstructed the time courses of cGMP hydrolysis in membrane preparations of rods and cones in response to corresponding light stimulation in the presence and absence of ATP with either GTP or GTPγS (*a*, 0.0085% in rods and *b*, 0.25% in cones; lines in Fig. 4A). The model also well simulated light intensity-dependent PDE activities (lines in Fig. 4B) with the rate constants for the Tr activation reactions (*k*_G1,0_–*k*_G1,3_ and *k*_G2_–*k*_G6_) as well as the reaction rates for VP phosphorylation and inactivation processes with and without ATP in both types of photoreceptors.

### Statistical examination of the model parameters

Mathematical models of biological system are demanded by the engineering field to utilize them in the medical and pharmacological area, thus the model parameter validation is important, although, historically they are continuously improved, if we look at the other type of cell models.

We first tried to manually adjust model parameters to reproduce simulation results which lies within the error bars of the reported wet experiments^15^ of front-end part of phototransduction, actually for Figs. 2A,B, 3A,B of both rods and cones. However, as we can see in Fig. 2, we could not reproduce results within the error bars of wet experiments as far as we tried, probably due to the error in the wet experiments.

Thus, we next tried to examine whether the parameter values with which the similar results are reproduced are unique or not based on the concept of Approximate Bayesian Computation^58^. Here we refer to the simulation results shown in Figs. 2 and 3 as “control results”. The examination was performed to find the randomized parameters with which similar results with control results are reproduced. We selected 21 parameters (*k*_RK1_, *k*_RK2_, *k*_RK3_, *k*_RK4_, *k*_RK5_, *k*_RK6_, *k*_RKi_, *k*_1_, *k*_G1_, *k*_G2_, *k*_G3_, *k*_G4_, *k*_G5_, *k*_G6_, *k*_G7_, *k*_P1_, *k*_P2_, *k*_P3_, *k*_P4_, *k*_decay_, *k*_Inact_) which are included in the front-end part of the phototransduction and can be considered to have effect to the front-end part results, while k2, k3, k4 were omitted since they have relatively small values and considered to have small effect to the results (see “Discussion” and Fig. S1 for sensitivity analysis results). For each of one statistical trial, we have generated 21 scaling factors in uniform random distribution in the log linear scale which ranges between 0.1 (${=10}^{-1}$) to 10.0 (${=10}^{+1}$). A set of simulation results corresponding to the simulation curves in Figs. 2, 3 were generated with the parameters multiplied by the generated 21 random scaling factors. The generated simulation results were evaluated by calculating the differences from the control data values shown as simulation results in Figs. 2A,B, 3A (+ATP and –ATP conditions), B (+ATP and –ATP conditions). The differences were calculated at the time points or flash intensity points used in wet experiments, for example, phosphorylation per VP* was evaluated at 2.5, 5, 7.5, 10 and 20 s time points for the evaluation of Fig. 2Aa rod case. The number of trials were 784,498,194 for rods, and 1,009,435,326 for cones. The similar results were selected with the criteria that all the evaluation results were within 30% of the control results, where all evaluation corresponds to the experiments of Pi/VP* vs time (Fig. 2A), Pi/VP* vs flash intensity (Fig. 2B), Tr/VP* vs time for +ATP and –ATP (Fig. 3A), and Tr/VP* vs flash intensity for +ATP and –ATP (Fig. 3B). The number of selected trials were 3970 for rods, and 1860 for cones.

**Figure 6:**
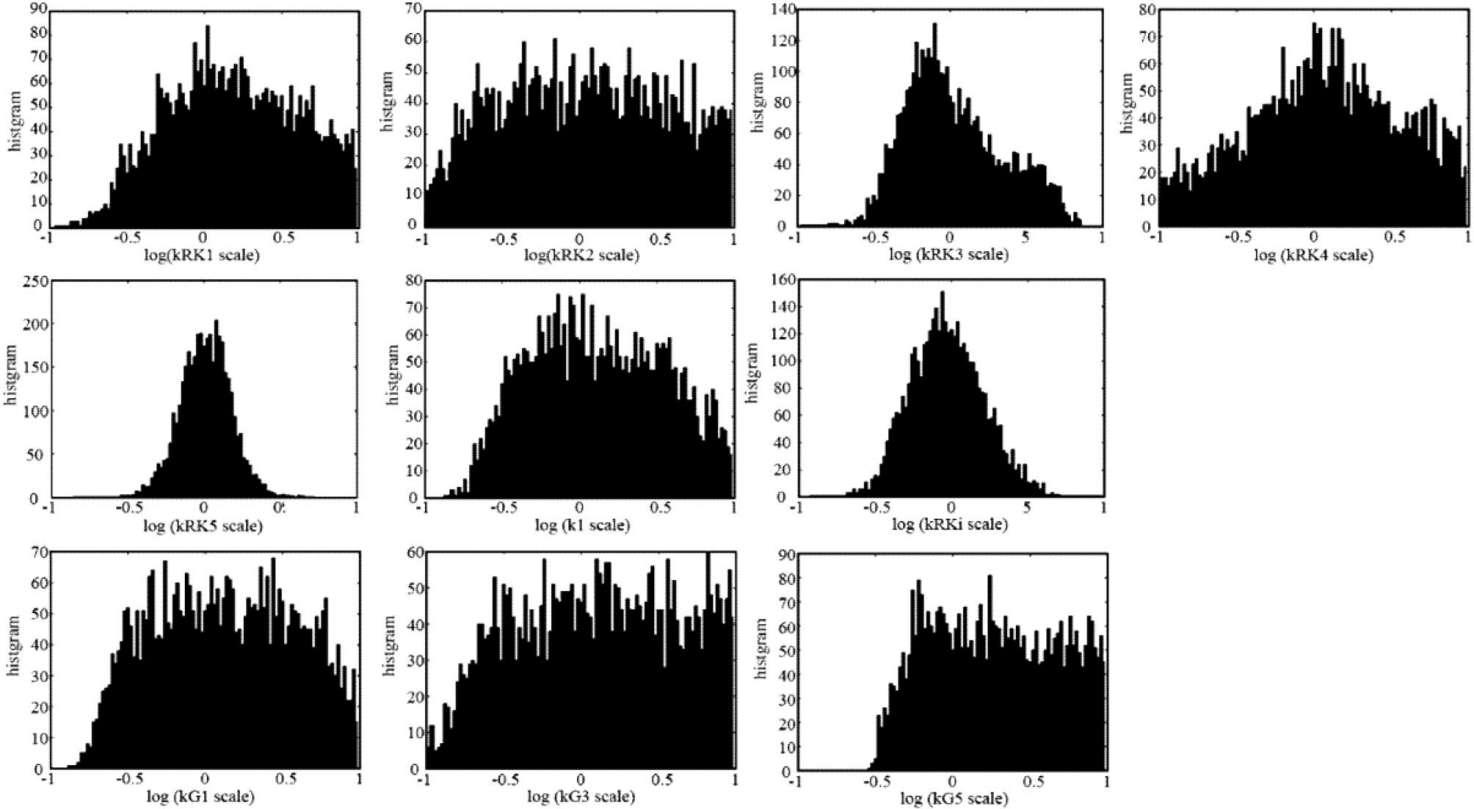
Histogram of phosphoryration related parameters of random test in rods. Histogram of phosphorylation related parameters of random test in rods are shown. The random test was performed by generating 21 uniform log linear distribution random number between 0.1 to 10.0 for phosphorylation related parameters (*k*_RK1_, *k*_RK2_, *k*_RK3_, *k*_RK4_, *k*_RK5_, *k*_RK6_, *k*_RKi_, *k*_1_, *k*_G1_, *k*_G2_, *k*_G3_, *k*_G4_, *k*_G5_, *k*_G6_, *k*_G7_, *k*_P1_, *k*_P2_, *k*_P3_, *k*_P4_, *k*_decay_, *k*_Inact_) and multiplied them to each parameter, and the resulting simulation data were evaluated by calculating difference between the control simulation data produced by the parameters in Table 3. This trial was performed for more than 780 M times, and the parameter set which produced results within 30% of the control data were selected (3970 trials), and the histogram of the random scale were plotted in the figure. Note that the parameters which showed concentrated distributions were selected and shown in the figure.

**Figure 7:**
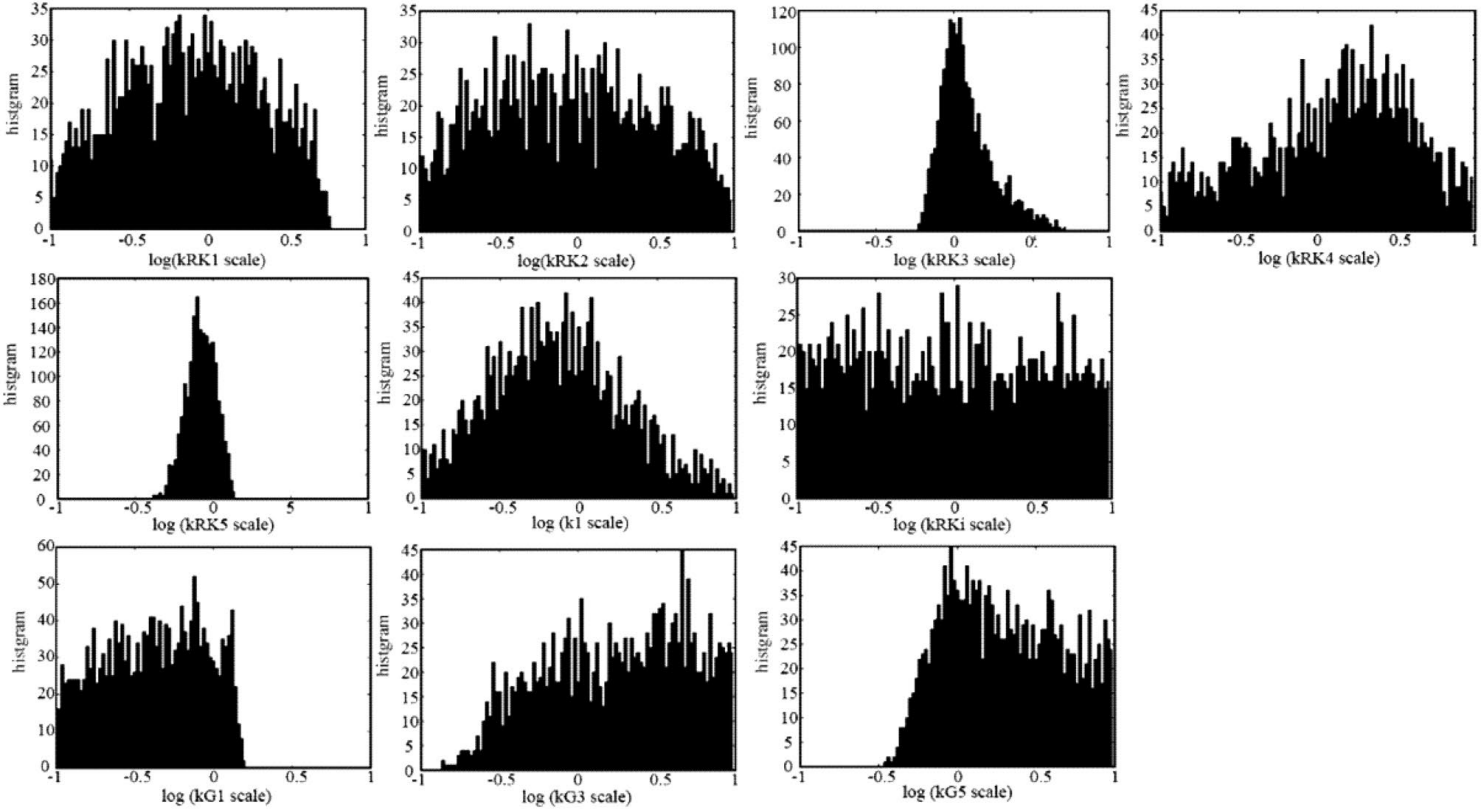
Histogram of phosphoryration related parameters of random test in cones. Histogram of phosphorylation related parameters of random test in cones are shown. See Fig. 6 caption for the experimental detail. The process was same with rods except the number of trials was more than 1G times, and the selected trial number was 1860.

Finally, for the selected results, the histogram (i.e. posterior distribution) of the parameter’s scaling factors which showed concentrated distribution in rods or cones were plotted with log scale in x-axis. The parameters were *k*_RK1_, *k*_RK3_, *k*_RK4_, *k*_RK5_, *k*_RKi_, *k*_1_, *k*_G1_, *k*_G3_ and *k*_G5_, and their histogram of rods and cones are shown in Figs. 6 and 7, respectively. In rod case (Fig. 6), *k*_RK3_, *k*_RK5_ and *k*_RKi_ showed narrow peak at scale factor of 1.0 (0.0 in log scale) and *k*_1_ had no distribution below 0.15 (− 0.8 in log scale), while the other parameters showed nearly flat distribution around or near scale factor of 1.0. In cone case (Fig. 7), *k*_RK3_, *k*_RK5_ showed narrow distribution, while *k*_1_ showed broad distribution compared to rod, and *k*_RKi_ showed flat distribution. The results may suggest that at least the parameters showing concentrated distribution may be necessary to be the values shown in Table 3. Also the *k*_1_ distribution in rod strongly suggests that the hypothetical intermediate state MII^i^ was necessary to reproduce the biochemical results, since *k*_1_ represents the rate constant of conversion from MII_n_ to hypothetical intermediate state MII_n_^i^. However for cones, the necessity of MII^i^ was not clear compared to rods, while the narrow peak of the *k*_1_ histogram of cones may suggest that the MII^i^ was also necessary in cones.

Since the target data were not the experimentally obtained ones but a simulation data, this results does not confirm that the proposed parameter values are close to the physiological true values. However, from this result, we can say that if we make a set of hypothetical experimental curves as shown in Figs. 2 and 3, we can say that the curves can be reproduced by set of these parameters within narrow range.

### Validating the phototransduction system by generating *I*_CNG_

As explained in the “Methods” section, the proposed phototransduction system must be compatible with the *I*_CNG_ generation system, but the system is very large so that the whole model parameter validation was not possible in this paper. Thus, in this paper, we restricted our validation by presenting at least one set of parameters of *I*_CNG_ generating system was available for rod. Kawamura et al.^14^ reported [GTP]_i_-dependent *I*_CNG_ recorded from a truncated outer segment of rods (Fig. 5A, circle). Physiologically, the basal [GTP]_i_ and [cGMP]_i_ in the dark are 1 mM and 2 μM, respectively, and the corresponding *I*_CNG_ are ~ − 4 pA in rods. Photoreceptor cells are thus slightly depolarized (− 40 mV^4^). Upon stimulation of the visual pigments, a decrease in the concentration of [cGMP]_i_ deactivates the CNG channels, reducing *I*_CNG_ to ~ 0 pA in response to a strong light stimulus. Kawamura and Tachibanaki reported that light-dependent changes in *I*_CNG_ recorded from outer segments of rods (Fig. 5B, solid lines)^8^. The intact elements of the current model simulated not only the [GTP]_i_-dependent *I*_CNG_ in rods in the dark (Fig. 5A, solid line) but also light-dependent *I*_CNG_ responses in the outer segments of rods (Fig. 5B). Note that these cGMP-dependent currents were simulated under voltage clamp condition (see Eq. S83) since the proposed model does not include other membrane current to calculate changes in membrane potentials^59^.

## Discussion

Retinal photoreceptor cells, rods and cones, convert photons of light into chemical and electrical signals as the first step in the visual transduction cascade. The chemical processes of the phototransduction system are very similar to each other in these photoreceptors. The light sensitivity and time resolution of the photoresponse in rods, however, are functionally different from those in cones. To quantitatively and systematically investigate how light intensity-dependent photoresponses are divergently regulated in rods and cones, a detailed mathematical models of the visual signal transduction system in these photoreceptors was developed where the front-end part was based on the Hamer model^10^. The current model successfully reconstructed a wide variety of light intensity-, ATP- and GTP-dependent changes in the concentrations and activities of phosphorylated VPs and activated Trs/PDEs in rods and cones^1,2,8,14,17,18,20,32,35^, when taking into account the localized molecular environment.

Compared to that in rods, the lower light sensitivity in cones was, at least in part, attributed to the lower affinity of the activated VP for Tr given that the concentration, as well as spatial distributions of VP and Tr at membranous disks in these photoreceptor cells, were very similar to each other. In experiments, the expression of cone Tr (G_α_ subunit) in rods decreased the light sensitivity of rods and the rate of Tr activation^9,33^. This biochemical study indicated that the molecular nature of the Tr G_α_ subunit in cones, not VP, contributes to the lower light sensitivity and response kinetics of Tr. Affinity difference is also discussed by analyzing the *I*_CNG_ current with simplified phototransduction system for mouse^13^, where the affinity difference is predicted as ~ 5, which is close to our model difference of 4.0.

Faster desensitization of activated VP through phosphorylation and thermal decay (inactivation) is another possible characteristic leading to lower light sensitivity in cones. While, properties of faster VP desensitization also contribute to the higher time resolution for phototransduction in cones. During the inactivation of MII^*^_0_, the functional intermediate state of inactive MII, MII^i^, was assumed before MII^*^ undergoing a complete transition to MIII in both types of photoreceptors^31^. Since the transition from MII^*^_0_ to MIII was suggested to be an event on the order of a few minutes in vitro^30,60^, considering that the state MII^i^ is indispensable to the termination of visual pigment activity: within ~ 10 s in rods and ~ 1 s in cones (see Fig. 3Aa and b, respectively).

In cones, the manually adjusted parameters of MII^*^ inactivation to reproduce experimental data was ~ 120-fold faster than that was in rods. The molecular nature of VP in cones (cone opsin), however, was indistinguishable from that in rods (rhodopsin), at least in terms of their light sensitivity in expression systems^9,33^. The mechanisms underlying the faster inactivation of cone opsin may be due to other environmental factors and need to be further explored in future experimental studies.

On the other hand, to reproduce continuing phosphorylation of VP even after complete termination of its activity, the phosphorylation rate constants for MII^i^ (*k*^i^_RK1_–*k*^i^_RK6_) were assumed to be slower (1/2) than those for MII^*^.

Deactivation of MII^*^ by the phosphorylation of RK, arrestin binding and also by the self-deactivation was predicted as ~ 2 times faster in cones in^13^. In our model, these processes were separately modeled, and the self-deactivation rate *k*_1_ showed 120 fold faster in cones. As explained above, this faster deactivation was essential to reproduce both phosphorylation time course shown in Fig. 2A, and transducin activation in –ATP condition shown in Fig. 3A. Since the self-deactivation rate is not separately determined in^13^, we could not directly compare with other reports or estimations, but since the balance between the three deactivation process are not clear, our model may not conflict with^13^.

When complete abolition of VP activities, within ~ 10 s after stimulus, PDE-dependent cGMP hydrolysis still progressed for ~ 30 s in rods and cones (shown as +ATP/+GTP in Fig. 4Aa and b, respectively) due to a longer lifetime of Tr* in the in vitro experimental systems. The assumption of faster inactivation of Tr* by PDE was therefore indispensable to obtain a faster recovery of *I*_CNG_, which was recorded in vivo (Fig. 5). In the current study, the mechanism underlying the fast inactivation of Tr* was attributed to the RGS9-mediated reaction, which was presumed to be intact under physiological conditions.

For the simulation experiments, the molecular environment of the membranous disk, where all the chemical reactions for phototransduction take place to generate *I*_CNG_ in vivo (Fig. 5), was assumed to be identical to that in the in vitro system (see Figs. 2, 3, 4 and Table 3). This assumption was based on the observations that VP, Tr, PDE, and RK are closely associated with membranous disks even after undergoing isolation procedures for the biochemical experiments^2^. If chemical reactions among freely diffusing factors are assumed, then the time course of phototransduction would be significantly slower due to the apparent reduction in the concentrations of key signaling molecules in the visual transduction cascade. The assumption made for the microdomain of the membranous disk seems to be verified since VP phosphorylation, Tr activation, and cGMP hydrolysis observed at different concentrations of phototransduction factors in different experimental studies were all well reconstructed by the current simulation study.

For the current model, most of the parameters were manually determined based on the experimental reports as fully described in the “Methods” or “Results” section. The rate constants for the reactions of Tr activation, *k*_G1,1_–*k*_G7_ (see Table 3), were estimated by model fitting to the initial rate of Tr activation in response to light flash stimulation as well as light intensity-dependent activation of Tr in vitro^18,32^ (see Fig. 3). However, the simulation experiments revealed that the time course and the light intensity-dependent activation of Tr, as shown in Fig. 3, may be reproduced with various sets of *k*_G1,0_–*k*_G7_ values. Specifically, the higher light sensitivity of rods can be reproduced even when the affinity of the activated VP to Tr ratio is lower than it is in cones when the subsequent molecular reactions (*k*_G1,1_–*k*_G7_) are faster. Since these parameters have not been conclusively determined by experimental studies, *k*_G1,1_–*k*_G7_ were set by referring to former simulation studies^10–12^. Furthermore, the G_α_-GTP-binding rate in MII^*^_0_–MII^*^_3_ (*k*_G1,0_–*k*_G1,3_) was assumed to decrease with successive phosphorylation of the VPs^34^. The reduction rate was determined by the parameter denoted by ω in R.3.6 (see the “Methods” section under “Activation and inactivation of transducins”). The current simulation study clarified that the higher the values of ω, the slower the transducin activation (Fig. 3) as well as PDE-mediated cGMP hydrolysis (Fig. 4) in the presence of ATP. Although the value of ω for rods was estimated to be 0.6 by Gibson et al.^34^, the value for cones was not given in the literature. In this study, ω was estimated by fitting the experimental data, as shown in Figs. 3 and 4, and found to be 0.9 for cones.

The reproduction of a single photon response^61^ has long been considered one of the most important characteristics to reproduce by a mathematical model. Prior theoretical analysis has concluded that the VP phosphorylation process requires multiple steps, and VP affinity for RK and Tr must exponentially decline as VP phosphorylation proceeds^10,11,15^. However, these theoretical models failed to reproduce the time courses of VP phosphorylation or Tr activation observed in the experiments, therefore, some unknown mechanisms must be involved to control these chemical processes. Importantly, the unknown mechanisms do not contradict the current model, since the model well reproduce both the microscopic and macroscopic experimental observations, although the current model failed to reproduce SPR experiments.

Validation model of *I*_CNG_ generation is still very elementary, thus more detailed model must be used to validate the proposed phototransduction model by using recent parameter values. However, this validation showed at least the phototransduction system is not too far from the realistic model since it could reproduce similar *I*_CNG_ current with elementary *I*_CNG_ generation model.

Meanwhile, other chemical reactions, listed below, were purely driven from assumptions due to limited details in literature. These model assumptions would derive new working hypothesis for future experimental studies to clarify corresponding uncertainties.

1. During the inactivation of MII^*^_0_, intermediate state of inactive MII, MII^i^, that has no capability to activate Tr, was introduced.
2. Elution of Gα-GTP, assumed for the in vitro model was essential to reproduce experimental data.
3. Reactions at membranous disk of the in vitro model needed to be evaluated after scaling substrate concentration.

As the model includes many parameters, the significance of each parameter to the simulation results are largely different. In order to analyze the effect of model parameters to the simulation results, sensitivity analysis was performed. 28 parameters related with VP phosphorylation, Tr activation and inactivation, PDE activation, GC activity, RGS activity were increased by 5% and the sum of squared differences between the resulting time courses of Pi/VP* and *I*_CNG_, and the original time courses were evaluated which are shown in Fig. S1A,B (in Supplement), respectively (results normalized). Figure S1A shows that the phosphorylation process was strongly related to the *k*_RK1_–*k*_RK6_ parameters, however, especially for cone, they were also related with Tr activation and inactivation, and PDE activation processes. Similarly, Fig. S1*B* shows that the *I*_CNG_ were strongly related with both phosphorylation, Tr activation and cGMP production processes. To see the detail of the relation between these parameters and the rising phase and the falling phase of *I*_CNG_, sensitivity analysis on the maximum and the minimum slope of *I*_CNG_ were performed (see Fig. S2 in Supplement). The rising phase was closely related with phosphorylation, Tr activation and PDE activation parameters (Fig. S2A), while the falling phase was related with phosphorylation, Tr activation and cGMP production and RGS activities (Fig. S2B).

### Limitations

Although the photocurrent production part of the model well demonstrated the rod *I*_CNG_ in response to light stimulation detailed model concerning calcium handling and membrane currents and also model for cones are required for the further analysis of the *I*_CNG_ production part.

## Supplementary Information

Supplementary Information.
