# Supplementary material for: Mathematical analysis of phototransduction reaction parameters in rods and cones
Source: Sci Rep. 2022 Nov 14;12:19529. doi: 10.1038/s41598-022-23069-0 (PMC9663442; doi:10.1038/s41598-022-23069-0)
Supplement: Supplementary file 1 — Supplementary Information. [file 41598_2022_23069_MOESM1_ESM.pdf]

Supplementary materials

Figure S1 Sensitivity analysis of VP phosphorylation and ICNG

A

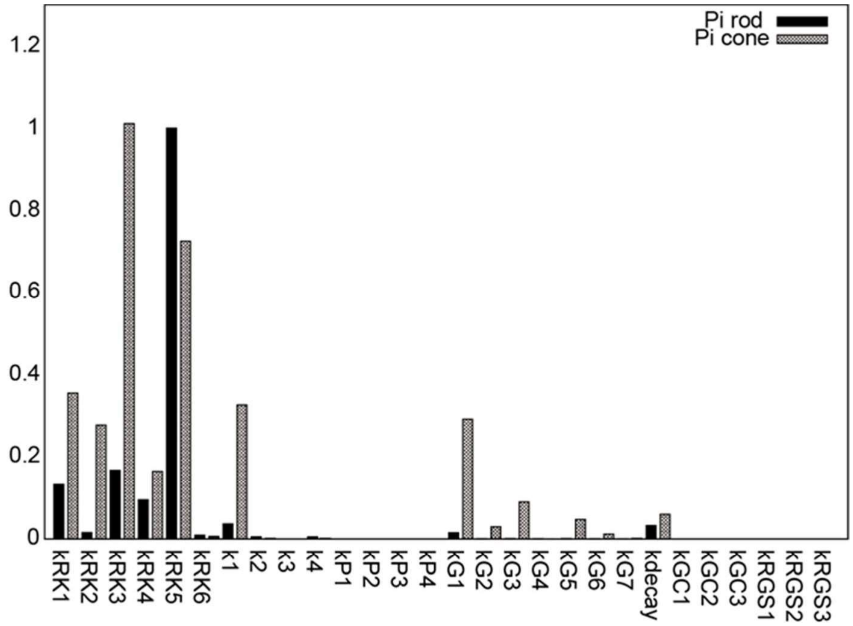

B

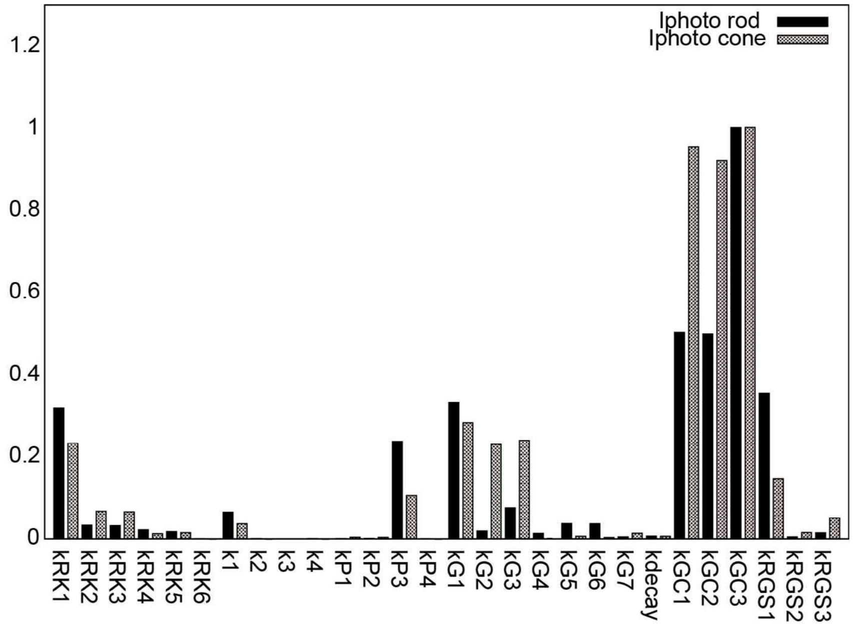

Results of sensitivity analysis to evaluate the impacts of model parameters to VP phosphorylation and ICNG. The figure indicates relative (sums of squared) differences in (A) VP phosphorylation and (B) ICNG (Figure 2Aa,b and5Ba,b, respectively) when parameter values (shown in Table 2) are increased by 5%, in comparison to control results.

Figure S2 Sensitivity analysis of rising and falling phase of ICNG

A

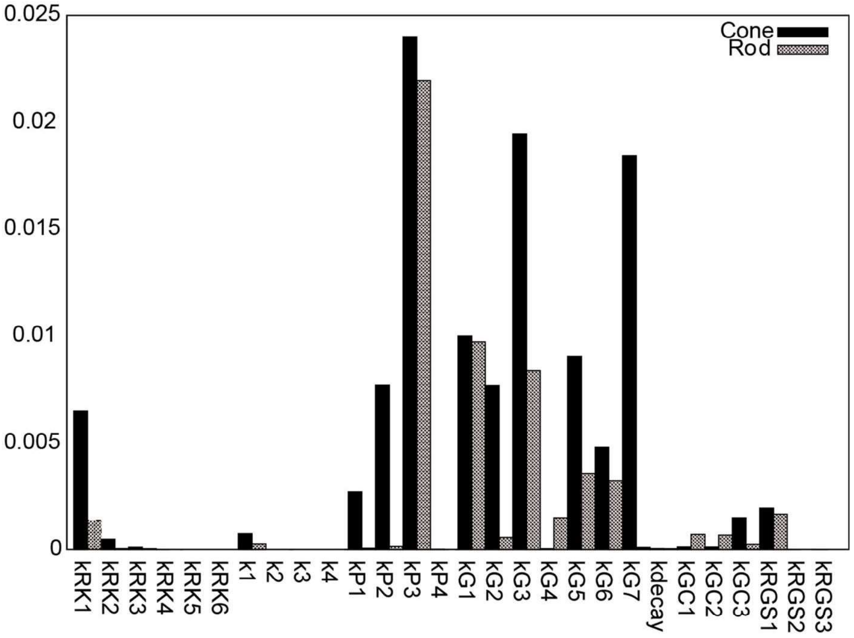

B

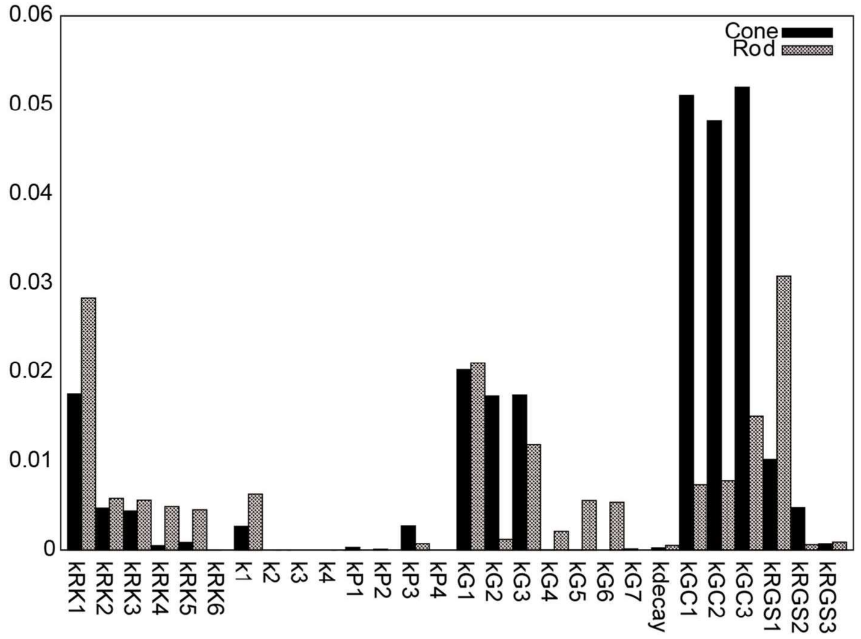

Results of sensitivity analysis to evaluate the impacts of model parameters to the rising and the falling phase of ICNG. The figure indicates relative (sums of squared) differences in (A) maximum and (B) minimum of dICNG/dt (Figure 5Bab, respectively) when parameter values (shown in Table 2) are increased by 5%, in comparison to control results.

Equations

in vitro

$$\frac{d}{dt}[MII_0^*] = -k_{RK1}[RK_{free}][MII_0^*] + k_{RK2}[MII_0^* \bullet RK_{pre}] - (k_1 + k_2 + k_4)[MII_0^*] - k_{G1,0}[MII_0^*][T_{free}] + k_{G2}[MII_0^* \bullet G \cdot GDP] + k_{G6}[MII_0^* \bullet G \cdot GTP] \quad \text{(Eq. S1)}$$

$$\frac{d}{dt}[MII_1^*] = -k_{RK1}[RK_{free}][MII_1^*] + k_{RK2}[MII_1^* \bullet RK_{pre}] + k_{RK6}[MII_1^* \bullet RK_{post}] - k_1[MII_1^*] - k_{G1,1}[MII_1^*][T_{free}] + k_{G2}[MII_1^* \bullet G \cdot GDP] + k_{G6}[MII_1^* \bullet G \cdot GTP] \quad \text{(Eq. S2)}$$

$$\frac{d}{dt}[MII_2^*] = -k_{RK1}[RK_{free}][MII_2^*] + k_{RK2}[MII_2^* \bullet RK_{pre}] + k_{RK6}[MII_2^* \bullet RK_{post}] - k_1[MII_2^*] - k_{G1,2}[MII_2^*][T_{free}] + k_{G2}[MII_2^* \bullet G \cdot GDP] \quad \text{(Eq. S3)}$$

$$\frac{d}{dt}[MII_3^*] = k_{RK6}[MII_3^* \bullet RK_{post}] - k_1[MII_3^*] - k_{G1,3}[MII_3^*][T_{free}] + k_{G2}[MII_3^* \bullet G \cdot GDP] \quad \text{(Eq. S4)}$$

$$\frac{d}{dt}[MII_0^* \bullet RK_{pre}] = k_{RK1}[RK_{free}][MII_0^*] - (k_{RK2} + k_{RK3}[ATP])[MII_0^* \bullet RK_{pre}] + k_{RK4}[MII_0^* \bullet RK_{pre} \cdot ATP] \quad \text{(Eq. S5)}$$

$$\frac{d}{dt}[MII_1^* \bullet RK_{pre}] = k_{RK1}[RK_{free}][MII_1^*] - (k_{RK2} + k_{RK3}[ATP])[MII_1^* \bullet RK_{pre}] + k_{RK4}[MII_1^* \bullet RK_{pre} \cdot ATP] \quad \text{(Eq. S6)}$$

$$\frac{d}{dt}[MII_2^* \bullet RK_{pre}] = k_{RK1}[RK_{free}][MII_2^*] - (k_{RK2} + k_{RK3}[ATP])[MII_2^* \bullet RK_{pre}] + k_{RK4}[MII_2^* \bullet RK_{pre} \cdot ATP] \quad \text{(Eq. S7)}$$

$$\frac{d}{dt}[MII_0^* \bullet RK_{pre} \cdot ATP] = k_{RK3}[ATP][MII_0^* \bullet RK_{pre}] - (k_{RK4} + k_{RK5})[MII_0^* \bullet RK_{pre} \cdot ATP] \quad \text{(Eq. S8)}$$

$$\frac{d}{dt}[MII_1^* \bullet RK_{pre} \cdot ATP] = k_{RK3}[ATP][MII_1^* \bullet RK_{pre}] - (k_{RK4} + k_{RK5})[MII_1^* \bullet RK_{pre} \cdot ATP] \quad \text{(Eq. S9)}$$

$$\frac{d}{dt}[MII_2^* \bullet RK_{pre} \cdot ATP] = k_{RK3}[ATP][MII_2^* \bullet RK_{pre}] - (k_{RK4} + k_{RK5})[MII_2^* \bullet RK_{pre} \cdot ATP] \quad \text{(Eq. S10)}$$

$$\frac{d}{dt}[MII_1^* \bullet RK_{post}] = k_{RK5}[MII_0^* \bullet RK_{pre} \cdot ATP] - k_{RK6}[MII_1^* \bullet RK_{post}] \quad \text{(Eq. S11)}$$

$$\frac{d}{dt}[MII_2^* \bullet RK_{post}] = k_{RK5}[MII_1^* \bullet RK_{pre} \cdot ATP] - k_{RK6}[MII_2^* \bullet RK_{post}] \quad \text{(Eq. S12)}$$

$$\frac{d}{dt}[MII_3^* \bullet RK_{post}] = k_{RK5}[MII_2^* \bullet RK_{pre} \cdot ATP] - k_{RK6}[MII_3^* \bullet RK_{post}] \quad \text{(Eq. S13)}$$

$$\frac{d}{dt}[MII_0^i] = k_1[MII_0^*] - k_{RK1}^i[RK_{free}][MII_0^i] + k_{RK2}^i[MII_0^i \bullet RK_{pre}] - (k_2 + k_4)[MII_0^i] \quad \text{(Eq. S14)}$$

$$\frac{d}{dt}[MII_1^i] = k_1[MII_1^*] - k_{RK1}^i[RK_{free}][MII_1^i] + k_{RK2}^i[MII_1^i \bullet RK_{pre}] + k_{RK6}^i[MII_1^i \bullet RK_{post}] \quad \text{(Eq. S15)}$$

$$\frac{d}{dt}[MII_2^i] = k_1[MII_2^*] - k_{RK1}^i[RK_{free}][MII_2^i] + k_{RK2}^i[MII_2^i \bullet RK_{pre}] + k_{RK6}^i[MII_2^i \bullet RK_{post}] \quad \text{(Eq. S16)}$$

$$\frac{d}{dt}[MII_3^i] = k_1[MII_3^*] + k_{RK6}^i[MII_3^i \bullet RK_{post}] \quad \text{(Eq. S17)}$$

$$\frac{d}{dt}[MII_0^i \bullet RK_{pre}] = k_{RK1}^i[RK_{free}][MII_0^i] - (k_{RK2}^i + k_{RK3}^i[ATP])[MII_0^i \bullet RK_{pre}] + k_{RK4}^i[MII_0^i \bullet RK_{pre} \cdot ATP] \quad \text{(Eq. S18)}$$

$$\frac{d}{dt}[MII_1^i \bullet RK_{pre}] = k_{RK1}^i[RK_{free}][MII_1^i] - (k_{RK2}^i + k_{RK3}^i[ATP])[MII_1^i \bullet RK_{pre}] + k_{RK4}^i[MII_1^i \bullet RK_{pre} \cdot ATP] \quad \text{(Eq. S19)}$$

$$\frac{d}{dt}[MII_2^i \bullet RK_{pre}] = k_{RK1}^i[RK_{free}][MII_2^i] - (k_{RK2}^i + k_{RK3}^i[ATP])[MII_2^i \bullet RK_{pre}] + k_{RK4}^i[MII_2^i \bullet RK_{pre} \cdot ATP] \quad \text{(Eq. S20)}$$

$$\frac{d}{dt}[MII_0^i \bullet RK_{pre} \cdot ATP] = k_{RK3}^i[ATP][MII_0^i \bullet RK_{pre}] - (k_{RK4}^i + k_{RK5}^i)[MII_0^i \bullet RK_{pre} \cdot ATP] \quad \text{(Eq. S21)}$$

$$\frac{d}{dt}[MII_1^i \bullet RK_{pre} \cdot ATP] = k_{RK3}^i[ATP][MII_1^i \bullet RK_{pre}] - (k_{RK4}^i + k_{RK5}^i)[MII_1^i \bullet RK_{pre} \cdot ATP] \quad \text{(Eq. S22)}$$

$$\frac{d}{dt}[MII_2^i \bullet RK_{pre} \cdot ATP] = k_{RK3}^i[ATP][MII_2^i \bullet RK_{pre}] - (k_{RK4}^i + k_{RK5}^i)[MII_2^i \bullet RK_{pre} \cdot ATP] \quad \text{(Eq. S23)}$$

$$\frac{d}{dt}[MII_1^i \bullet RK_{post}] = k_{RK5}^i[MII_0^i \bullet RK_{pre} \cdot ATP] - k_{RK6}^i[MII_1^i \bullet RK_{post}] \quad \text{(Eq. S24)}$$

$$\frac{d}{dt}[MII_2^i \bullet RK_{post}] = k_{RK5}^i[MII_1^i \bullet RK_{pre} \cdot ATP] - k_{RK6}^i[MII_2^i \bullet RK_{post}] \quad \text{(Eq. S25)}$$

$$\frac{d}{dt}\left[MII_3^i \bullet RK_{post}\right] = k_{RK5}^i\left[MII_2^i \bullet RK_{pre} \cdot ATP\right] - k_{RK6}^i\left[MII_3^i \bullet RK_{post}\right] \quad \text{(Eq. S26)}$$

$$\frac{d}{dt}[MIII] = k_2\left([MII_0^*] + \left[MII_0^i\right]\right) - k_3[MIII] \quad \text{(Eq. S27)}$$

$$\frac{d}{dt}[retinal] = k_4\left([MII_0^*] + \left[MII_0^i\right]\right) + k_3[MIII] \quad \text{(Eq. S28)}$$

$$\frac{d}{dt}[MII_0^* \bullet G \cdot GDP] = k_{G1,0}[MII_0^*]\left[T_{free}\right] - (k_{G2} + k_{G3})[MII_0^* \bullet G \cdot GDP] + k_{G4}[GDP][MII_0^* \bullet G] \quad \text{(Eq. S29)}$$

$$\frac{d}{dt}[MII_1^* \bullet G \cdot GDP] = k_{G1,1}[MII_1^*]\left[T_{free}\right] - (k_{G2} + k_{G3})[MII_1^* \bullet G \cdot GDP] + k_{G4}[GDP][MII_1^* \bullet G] \quad \text{(Eq. S30)}$$

$$\frac{d}{dt}[MII_2^* \bullet G \cdot GDP] = k_{G1,2}[MII_2^*]\left[T_{free}\right] - (k_{G2} + k_{G3})[MII_2^* \bullet G \cdot GDP] + k_{G4}[GDP][MII_2^* \bullet G] \quad \text{(Eq. S31)}$$

$$\frac{d}{dt}[MII_3^* \bullet G \cdot GDP] = k_{G1,3}[MII_3^*]\left[T_{free}\right] - (k_{G2} + k_{G3})[MII_3^* \bullet G \cdot GDP] + k_{G4}[GDP][MII_3^* \bullet G] \quad \text{(Eq. S32)}$$

$$\frac{d}{dt}[MII_0^* \bullet G] = k_{G3}[MII_0^* \bullet G \cdot GDP] - (k_{G4}[GDP] + k_{G5}[GTP])[MII_0^* \bullet G] \quad \text{(Eq. S33)}$$

$$\frac{d}{dt}[MII_1^* \bullet G] = k_{G3}[MII_1^* \bullet G \cdot GDP] - (k_{G4}[GDP] + k_{G5}[GTP])[MII_1^* \bullet G] \quad \text{(Eq. S34)}$$

$$\frac{d}{dt}[MII_2^* \bullet G] = k_{G3}[MII_2^* \bullet G \cdot GDP] - (k_{G4}[GDP] + k_{G5}[GTP])[MII_2^* \bullet G] \quad \text{(Eq. S35)}$$

$$\frac{d}{dt}[MII_3^* \bullet G] = k_{G3}[MII_3^* \bullet G \cdot GDP] - (k_{G4}[GDP] + k_{G5}[GTP])[MII_3^* \bullet G] \quad \text{(Eq. S36)}$$

$$\frac{d}{dt}[MII_0^* \bullet G \cdot GTP] = k_{G5}[GTP][MII_0^* \bullet G] - k_{G6}[MII_0^* \bullet G \cdot GTP] \quad \text{(Eq. S37)}$$

$$\frac{d}{dt}[MII_1^* \bullet G \cdot GTP] = k_{G5}[GTP][MII_1^* \bullet G] - k_{G6}[MII_1^* \bullet G \cdot GTP] \quad \text{(Eq. S38)}$$

$$\frac{d}{dt}[MII_2^* \bullet G \cdot GTP] = k_{G5}[GTP][MII_2^* \bullet G] - k_{G6}[MII_2^* \bullet G \cdot GTP] \quad \text{(Eq. S39)}$$

$$\frac{d}{dt}[MII_3^* \bullet G \cdot GTP] = k_{G5}[GTP][MII_3^* \bullet G] - k_{G6}[MII_3^* \bullet G \cdot GTP] \quad \text{(Eq. S40)}$$

$$\frac{d}{dt}[G \cdot GTP] = k_{G6}([MII_0^* \bullet G \cdot GTP] + [MII_1^* \bullet G \cdot GTP] + [MII_2^* \bullet G \cdot GTP] + [MII_3^* \bullet G \cdot GTP]) - k_{G7}[G \cdot GTP] \quad \text{(Eq. S41)}$$

$$\frac{d}{dt}[G_\alpha \cdot GTP] = k_{G7}[G \cdot GTP] - k_{P1}[PDE_{free}][G_\alpha \cdot GTP] - k_{decay}[G_\alpha \cdot GTP] - k_{elution}[G_\alpha \cdot GTP] \quad \text{(Eq. S42)}$$

$$\frac{d}{dt}[G_\alpha \cdot GTP_{eluted}] = k_{elution}[G_\alpha \cdot GTP] - k_{decay}[G_\alpha \cdot GTP_{eluted}] \quad \text{(Eq. S43)}$$

$$\frac{d}{dt}[PDE \bullet G_\alpha \cdot GTP] = k_{P1}[PDE_{free}][G_\alpha \cdot GTP] - k_{P2}[PDE \bullet G_\alpha \cdot GTP] - k_{decay}[PDE \bullet G_\alpha \cdot GTP] \quad \text{(Eq. S44)}$$

$$\frac{d}{dt}[PDE^* \bullet G_\alpha \cdot GTP] = k_{P2}[PDE \bullet G_\alpha \cdot GTP] - k_{decay}[PDE^* \bullet G_\alpha \cdot GTP] \quad \text{(Eq. S45)}$$

$$[RK_{free}] = [RK_{total}] - \left(\sum_{n=1}^{3}([MII_n^* \bullet RK_{post}] + [MII_n^i \bullet RK_{post}]) + \sum_{n=0}^{2}([MII_n^* \bullet RK_{pre}] + [MII_n^i \bullet RK_{pre}] + [MII_n^* \bullet RK_{pre} \cdot ATP] + [MII_n^i \bullet RK_{pre} \cdot ATP])\right) \quad \text{(Eq. S46)}$$

$$[PDE_{free}] = [PDE_{total}] - ([PDE \bullet G_\alpha \cdot GTP] + [PDE^* \bullet G_\alpha \cdot GTP]) \quad \text{(Eq. S47)}$$

$$[T_{free}] = [T_{total}] - ([MII_0^* \bullet G \cdot GDP] + [MII_0^* \bullet G] + [MII_0^* \bullet G \cdot GTP] + [MII_1^* \bullet G \cdot GDP] + [MII_1^* \bullet G] + [MII_1^* \bullet G \cdot GTP] + [MII_2^* \bullet G] + [MII_2^* \bullet G \cdot GTP] + [MII_3^* \bullet G] + [MII_3^* \bullet G \cdot GTP] + [G \cdot GTP] + [G_\alpha \cdot GTP] + [PDE \bullet G_\alpha \cdot GTP] + [PDE^* \bullet G_\alpha \cdot GTP] + [G_\alpha \cdot GTP_{eluted}]) \quad \text{(Eq. S48)}$$

$$[GTP_{free}] = [GTP_{total}] - ([MII_0^* \bullet G \cdot GTP] + [MII_1^* \bullet G \cdot GTP] + [MII_2^* \bullet G \cdot GTP] + [MII_3^* \bullet G \cdot GTP] + [G \cdot GTP] + [G_\alpha \cdot GTP] + [PDE \bullet G_\alpha \cdot GTP] + [PDE^* \bullet G_\alpha \cdot GTP] + [GC \cdot GTP]) \quad \text{(Eq. S49)}$$

$$\frac{d}{dt}[ATP] = \sum_{n=0}^{2}\left(k_{RK4}\left[MII_n^* \bullet RK_{pre} \cdot ATP\right] + k_{RK4}^{i}\left[MII_n^{i} \bullet RK_{pre} \cdot ATP\right]\right) - [ATP]\sum_{n=0}^{2}\left(k_{RK3}\left[MII_n^* \bullet RK_{pre}\right] + k_{RK3}^{i}\left[MII_n^{i} \bullet RK_{pre}\right]\right) \quad \text{(Eq. S50)}$$

intact

$$\frac{d}{dt}[MII_0^*] = -k_{RK1}\left[RK_{free}\right][MII_0^*] + k_{RK2}\left[MII_0^* \bullet RK_{pre}\right] - (k_1 + k_2 + k_4)[MII_0^*] - k_{G1,0}[MII_0^*]\left[T_{free}\right] + k_{G2}[MII_0^* \bullet G \cdot GDP] + k_{G6}[MII_0^* \bullet G \cdot GTP] \quad \text{(Eq. S51)}$$

$$\frac{d}{dt}[MII_1^*] = k_{RK6}\left[MII_1^* \bullet RK_{post}\right] - k_1[MII_1^*] \quad \text{(Eq. S52)}$$

$$\frac{d}{dt}\left[MII_0^* \bullet RK_{pre}\right] = k_{RK1}\left[RK_{free}\right][MII_0^*] - (k_{RK2} + k_{RK3}[ATP])\left[MII_0^* \bullet RK_{pre}\right] + k_{RK4}\left[MII_0^* \bullet RK_{pre} \cdot ATP\right] \quad \text{(Eq. S53)}$$

$$\frac{d}{dt}\left[MII_0^* \bullet RK_{pre} \cdot ATP\right] = k_{RK3}[ATP]\left[MII_0^* \bullet RK_{pre}\right] - (k_{RK4} + k_{RK5})\left[MII_0^* \bullet RK_{pre} \cdot ATP\right] \quad \text{(Eq. S54)}$$

$$\frac{d}{dt}\left[MII_1^* \bullet RK_{post}\right] = k_{RK5}\left[MII_0^* \bullet RK_{pre} \cdot ATP\right] - k_{RK6}\left[MII_1^* \bullet RK_{post}\right] \quad \text{(Eq. S55)}$$

$$\frac{d}{dt}\left[MII_0^{i}\right] = k_1[MII_0^*] - k_{RK1}^{i}\left[RK_{free}\right]\left[MII_0^{i}\right] + k_{RK2}^{i}\left[MII_0^{i} \bullet RK_{pre}\right] - (k_2 + k_4)\left[MII_0^{i}\right] \quad \text{(Eq. S56)}$$

$$\frac{d}{dt}\left[MII_1^{i}\right] = k_1[MII_1^*] + k_{RK6}^{i}\left[MII_1^{i} \bullet RK_{post}\right] \quad \text{(Eq. S57)}$$

$$\frac{d}{dt}\left[MII_0^{i} \bullet RK_{pre}\right] = k_{RK1}^{i}\left[RK_{free}\right]\left[MII_0^{i}\right] - \left(k_{RK2}^{i} + k_{RK3}^{i}[ATP]\right)\left[MII_0^{i} \bullet RK_{pre}\right] + k_{RK4}^{i}\left[MII_0^{i} \bullet RK_{pre} \cdot ATP\right] \quad \text{(Eq. S58)}$$

$$\frac{d}{dt}\left[MII_0^{i} \bullet RK_{pre} \cdot ATP\right] = k_{RK3}^{i}[ATP]\left[MII_0^{i} \bullet RK_{pre}\right] - \left(k_{RK4}^{i} + k_{RK5}^{i}\right)\left[MII_0^{i} \bullet RK_{pre} \cdot ATP\right] \quad \text{(Eq. S59)}$$

$$\frac{d}{dt}\left[MII_1^{i} \bullet RK_{post}\right] = k_{RK5}^{i}\left[MII_0^{i} \bullet RK_{pre} \cdot ATP\right] - k_{RK6}^{i}\left[MII_1^{i} \bullet RK_{post}\right] \quad \text{(Eq. S60)}$$

$$\frac{d}{dt}[MIII] = k_2([MII_0^*] + [MII_0^i]) - k_3[MIII] \quad \text{(Eq. S61)}$$

$$\frac{d}{dt}[retinal] = k_4([MII_0^*] + [MII_0^i]) + k_3[MIII] \quad \text{(Eq. S62)}$$

$$\frac{d}{dt}[MII_0^* \bullet G \cdot GDP] = k_{G1,0}[MII_0^*][T_{free}] - (k_{G2} + k_{G3})[MII_0^* \bullet G \cdot GDP] + k_{G4}[GDP][MII_0^* \bullet G] \quad \text{(Eq. S63)}$$

$$\frac{d}{dt}[MII_0^* \bullet G] = k_{G3}[MII_0^* \bullet G \cdot GDP] - (k_{G4}[GDP] + k_{G5}[GTP])[MII_0^* \bullet G] \quad \text{(Eq. S64)}$$

$$\frac{d}{dt}[MII_0^* \bullet G \cdot GTP] = k_{G5}[GTP][MII_0^* \bullet G] - k_{G6}[MII_0^* \bullet G \cdot GTP] \quad \text{(Eq. S65)}$$

$$\frac{d}{dt}[G \cdot GTP] = k_{G6}[MII_0^* \bullet G \cdot GTP] - k_{G7}[G \cdot GTP] \quad \text{(Eq. S66)}$$

$$\frac{d}{dt}[G_\alpha \cdot GTP] = k_{G7}[G \cdot GTP] - k_{P1}[PDE_{free}][G_\alpha \cdot GTP] - k_{decay}[G_\alpha \cdot GTP] \quad \text{(Eq. S67)}$$

$$\frac{d}{dt}[PDE \bullet G_\alpha \cdot GTP] = k_{P1}[PDE_{free}][G_\alpha \cdot GTP] - k_{P2}[PDE \bullet G_\alpha \cdot GTP] - k_{decay}[PDE \bullet G_\alpha \cdot GTP] \quad \text{(Eq. S68)}$$

$$\frac{d}{dt}[PDE^* \bullet G_\alpha \cdot GTP] = k_{P2}[PDE \bullet G_\alpha \cdot GTP] + (\beta_{sub} + k_{P4})[cG \cdot PDE^* \bullet G_\alpha \cdot GTP] - k_{P3}[PDE^* \bullet G_\alpha \cdot GTP][cGMP] - k_{decay}[PDE^* \bullet G_\alpha \cdot GTP] - k_{RGS1}[PDE^* \bullet G_\alpha \cdot GTP][RGS9_{free}] + k_{RGS2}[RGS9 \bullet PDE^* \bullet G_\alpha \cdot GTP] \quad \text{(Eq. S69)}$$

$$\frac{d}{dt}[cG \cdot PDE^* \bullet G_\alpha \cdot GTP] = k_{P3}[PDE^* \bullet G_\alpha \cdot GTP][cGMP] - (\beta_{sub} + k_{P4})[cG \cdot PDE^* \bullet G_\alpha \cdot GTP] \quad \text{(Eq. S70)}$$

$$\frac{d}{dt}[RGS9 \bullet PDE^* \bullet G_\alpha \cdot GTP] = k_{RGS1}[PDE^* \bullet G_\alpha \cdot GTP][RGS9_{free}] - (k_{RGS2} + k_{RGS3})[RGS9 \bullet PDE^* \bullet G_\alpha \cdot GTP] \quad \text{(Eq. S71)}$$

$$\frac{d}{dt}[GC\cdot GTP] = k_{GC1}[GC_{free}][GTP_{free}] - k_{GC2}[GC\cdot GTP] - \frac{k_{GC3}}{1+\left(\frac{[Ca^{2+}]}{K_c}\right)^m}[GC\cdot GTP] \quad \text{(Eq. S72)}$$

$$[RK_{free}] = [RK_{total}] - ([MII_1^* \bullet RK_{post}] + [MII_1^i \bullet RK_{post}] + [MII_0^* \bullet RK_{pre}] + [MII_0^i \bullet RK_{pre}] + [MII_0^* \bullet RK_{pre}\cdot ATP] + [MII_0^i \bullet RK_{pre}\cdot ATP]) \quad \text{(Eq. S73)}$$

$$[PDE_{free}] = [PDE_{total}] - ([PDE \bullet G_\alpha\cdot GTP] + [PDE^* \bullet G_\alpha\cdot GTP] + [cG\cdot PDE^* \bullet G_\alpha\cdot GTP] + [RGS9 \bullet PDE^* \bullet G_\alpha\cdot GTP]) \quad \text{(Eq. S74)}$$

$$[T_{free}] = [T_{total}] - ([MII_0^* \bullet G\cdot GDP] + [MII_0^* \bullet G] + [MII_0^* \bullet G\cdot GTP] + [G\cdot GTP] + [G_\alpha\cdot GTP] + [PDE \bullet G_\alpha\cdot GTP] + [PDE^* \bullet G_\alpha\cdot GTP] + [cG\cdot PDE^* \bullet G_\alpha\cdot GTP] + [RGS9 \bullet PDE^* \bullet G_\alpha\cdot GTP]) \quad \text{(Eq. S75)}$$

$$[RGS9_{free}] = [RGS9_{total}] - [RGS9 \bullet PDE^* \bullet G_\alpha\cdot GTP] \quad \text{(Eq. S76)}$$

$$[GC_{free}] = [GC_{total}] - [GC\cdot GTP] \quad \text{(Eq. S77)}$$

$$[GTP_{free}] = [GTP_{total}] - ([MII_0^* \bullet G\cdot GTP] + [G\cdot GTP] + [G_\alpha\cdot GTP] + [PDE \bullet G_\alpha\cdot GTP] + [PDE^* \bullet G_\alpha\cdot GTP] + [cG\cdot PDE^* \bullet G_\alpha\cdot GTP] + [RGS9 \bullet PDE^* \bullet G_\alpha\cdot GTP] + [GC\cdot GTP]) \quad \text{(Eq. S78)}$$

$$\frac{d}{dt}[ATP] = k_{RK4}[MII_0^* \bullet RK_{pre}\cdot ATP] + k_{RK4}^i[MII_0^i \bullet RK_{pre}\cdot ATP] - k_{RK3}[ATP][MII_0^* \bullet RK_{pre}] - k_{RK3}^i[ATP][MII_0^i \bullet RK_{pre}] \quad \text{(Eq. S79)}$$

$$\frac{d}{dt}[cGMP] = \frac{k_{GC3}}{1+\left(\frac{[Ca^{2+}]}{K_c}\right)^m}[GC\cdot GTP] - (\beta_{dark}[cGMP] + \beta_{sub}[cG\cdot PDE^* \bullet G_\alpha\cdot GTP]) \quad \text{(Eq. S80)}$$

$$\frac{d}{dt}[Ca^{2+}] = -b\frac{I_{photo}}{2F\cdot vol} - \gamma_{Ca}([Ca^{2+}] - c_0) - k_{b1}[Ca^{2+}](e_T - [Cab]) + k_{b2}[Cab] \quad \text{(Eq. S81)}$$

$$\frac{d}{dt}[Cab] = k_{b1}[Ca^{2+}](e_T - [Cab]) - k_{b2}[Cab] \quad \text{(Eq. S82)}$$

$$I_{photo} = G_{max}\frac{[cGMP]^{n_h}}{[cGMP]^{n_h} + K_m^{n_h}}(V_m - E_p) \quad \text{(Eq. S83)}$$
